# Discovery of SOCS7 as a versatile E3 ligase for protein-based degraders

**DOI:** 10.1016/j.isci.2024.109802

**Published:** 2024-04-23

**Authors:** Anaïs Cornebois, Marie Sorbara, Margot Cristol, Emmanuelle Vigne, Pierre Cordelier, Klervi Desrumeaux, Nicolas Bery

**Affiliations:** 1 Université de Toulouse, Inserm, CNRS, Université Toulouse III-Paul Sabatier, Centre de Recherches en Cancérologie de Toulouse, 31100 Toulouse, France; 2 Sanofi, Large Molecule Research, 94400 Vitry-sur-Seine, France

**Keywords:** Molecular biology, Cell biology, Cancer

## Abstract

Targeted protein degradation (TPD) strategy harnesses the ubiquitin-proteasome system (UPS) to degrade a protein of interest (POI) by bringing it into proximity with an E3 ubiquitin ligase. However, the limited availability of functional E3 ligases and the emergence of resistance through mutations in UPS components restrict this approach. Therefore, identifying alternative E3 ligases suitable for TPD is important to develop new degraders and overcome potential resistance mechanisms. Here, we use a protein-based degrader method, by fusing an anti-tag intracellular antibody to an E3 ligase, to screen E3 ligases enabling the degradation of a tagged POI. We identify SOCS7 E3 ligase as effective biodegrader, able to deplete its target in various cell lines regardless of the POI’s subcellular localization. We show its utility by generating a SOCS7-based KRAS degrader that inhibits mutant KRAS pancreatic cancer cells’ proliferation. These findings highlight SOCS7 versatility as valuable E3 ligase for generating potent degraders.

## Highlights

• A cell-based screening identifies SOCS7 as a potent E3 ligase amenable to TPD
• SOCS7-based biodegraders are active regardless of the cell type, POI localization
• The geometry and affinity of SOCS7-based biodegraders impact their activity
• A SOCS7-based KRAS degrader inhibits pancreatic cancer cells proliferation

## Graphical abstract

**Figure:**
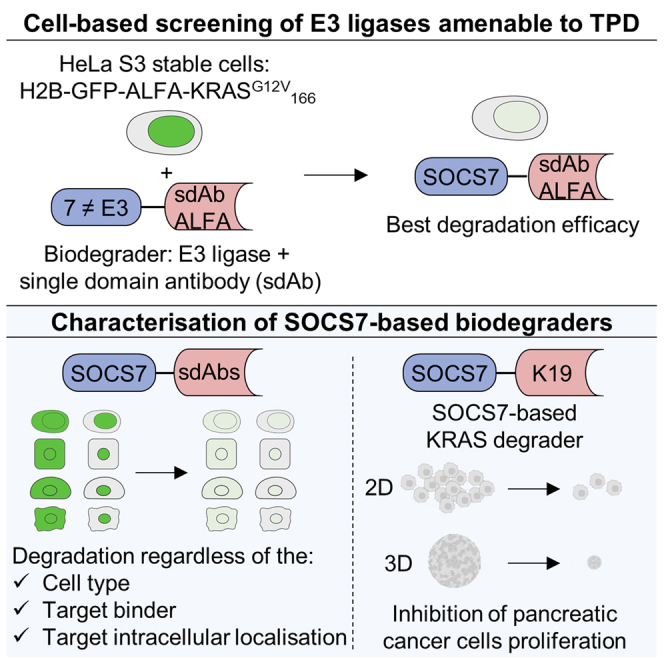

## Introduction

Targeted protein degradation (TPD) represents a promising approach in both therapeutic and research areas. Several TPD technologies have been established by hijacking the natural intracellular degradation pathways, including proteasome-dependent proteolysis.^1^ In this context, TPD brings into close proximity a protein of interest (POI) with an active E3 ubiquitin ligase leading to polyubiquitination and subsequent degradation of the POI by the proteasome. Two main categories of such degraders have been developed: small molecule- and protein-based degraders. Small molecule-based degraders, also known as proteolysis-targeting chimeras (PROTACs), connect a POI-specific ligand and an E3 ligase-specific compound via a chemical linker.^1^^,^^2^ These degraders hold great promise for therapy and many have already reached clinical trials.^3^ However, the development of functional PROTACs remains challenging. To generate potent PROTACs, several rounds of optimization are required, including identifying a suitable site for linker attachment on the protein ligand, determining the optimal linker length and composition, which all can be time consuming. In addition, PROTACs are limited by the availability of small molecule ligands targeting both the protein target and E3 ligase. Protein-based degraders, also known as bioPROTACs or biodegraders, consist of a fusion between a POI-specific protein binder (*e.g*., single domain antibodies (sdAbs),^4^^,^^5^^,^^6^^,^^7^^,^^8^ antibody mimetics,^8^^,^^9^ or peptides^10^^,^^11^) and an E3 ligase domain.^12^^,^^13^ The generation of functional biodegraders is facilitated not only by straightforward molecular biology processes but also by the relative ease to select intracellular protein binders that can theoretically bind any POI with high affinity and specificity. This includes hard-to-drug POIs such as post-translationally modified proteins,^14^ proteins with specific conformation^5^^,^^15^ or those involved in protein-protein interactions^8^^,^^9^^,^^16^ like transcription factors.^6^^,^^17^^,^^18^

While only a limited number of E3 ubiquitin ligases have been used for the development of small molecule- and protein-based degraders so far, the human genome is predicted to encode over 600 E3 ligases. In addition, nature has evolved this high number of E3 ligases to precisely control proteasomal degradation, including through by spatial and temporal regulation of their expression and activation.^19^ Hence, this restricts their usage for TPD to some cellular contexts (*e.g*., target or cell types), which limits the development of novel degraders.^20^^,^^21^ Furthermore, an increasing body of evidence suggests that the development of acquired resistance to various degraders is associated with genomic alterations in critical components of the ubiquitin-proteasome system (UPS).^22^^,^^23^^,^^24^ Hence, the discovery of functional and versatile E3 ligases is urgently needed to overcome these limitations. To do that, a few proximity-based screening methods, including the protein-based degrader system, have uncovered E3 ligases amenable to TPD.^11^^,^^21^^,^^25^ However, for an E3 ligase to be considered versatile for targeted degradation, it must meet several requirements that are usually not all tested downstream of the screening method. In particular, these include the capacity to degrade a target in different subcellular compartments,^26^ various cell types or organisms,^27^ and to be active with multiple POIs and POI binders.^11^ Obtaining such versatile E3 ligases for TPD would enhance the utility of biodegraders as biological tools but also augment their potential as therapeutic strategy.

We report here the identification of E3 ligases that can be recruited for TPD through the biodegrader system using a cell-based screening approach. We characterize the most potent E3 ligase, SOCS7, by comparing its activity as proximity-induced degrader of a POI, to other known E3 ligases used in TPD (e.g., the Von Hippel-Lindau [VHL]^28^ or speckle-type POZ protein [SPOP]^27^). We show SOCS7 is the most versatile E3 ligase because SOCS7-based biodegrader promotes the degradation of POIs within distinct subcellular localizations in multiple cell lines when fused to different POI binders. Additionally, we explore the impact of the geometry and sdAb’s affinity on the efficacy of SOCS7-based biodegrader degradation. Finally, we leverage these properties to construct a SOCS7-based KRAS degrader, that reduces RAS-dependent signaling and impedes the proliferation of pancreatic cancer cells.

## Results

### Setting up a tag-based system to screen E3 ligases for TPD

To determine whether an E3 ligase is amenable to intracellular antibody-based targeted degradation, we first designed a chimeric POI: GFP-ALFA-KRAS^G12V^_166_, for which intracellular binders are available for each component of this POI. GFP is used as fluorescent reporter to quantify the degradation of the chimeric POI by flow cytometry. The ALFA tag is a recent reported small size and versatile tag (13 amino acids), which has a cognate high affinity sdAb (26 pM, herein named sdAb ALFA).^29^ KRAS^G12V^_166_ is a G12V mutant of KRAS deleted of its 22 last carboxy-terminal amino acids and that does not bind to the plasma membrane.^16^ To complete this intracellular antibody-based targeted degradation system, we generated a non-binding sdAb as negative control (hereafter named sdAb control or sdAb Ctl). For this purpose, we analyzed the 3D structure of the sdAb ALFA/ALFA tag complex^29^ and mutated five key residues in the sdAb ALFA paratope (Figures S1A and S1B). These mutations successfully ablated the binding of sdAb Ctl to the ALFA tag compared to the parental sdAb ALFA as shown by bioluminescence resonance energy transfer (BRET) donor saturation assay (Figure S1C) and co-immunoprecipitation experiment (Figure S1D).

**Figure 1:**
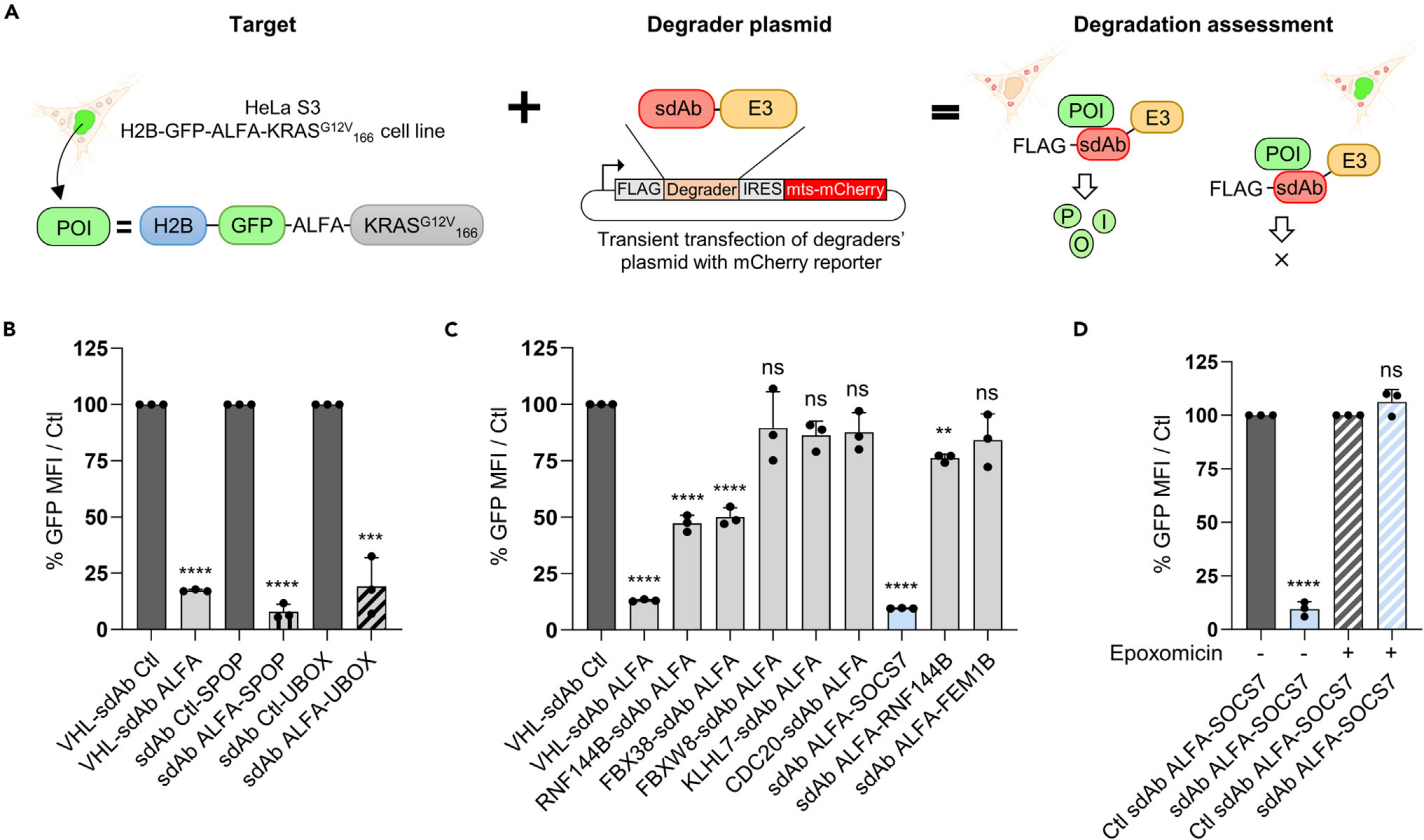
Identification of SOCS7 as potent E3 ligase amenable to targeted protein degradation (A) General scheme of the degradation screening method. HeLa S3 cells stably express H2B-GFP-ALFA-KRAS^G12V^_166_ chimeric POI. Histone H2B is for the nuclear localization (chromatin anchorage) of the POI, GFP is used as detection protein, ALFA tag is the binding protein of sdAb ALFA and KRAS^G12V^_166_ is a truncated version of mutant KRAS^G12V^ deleted of its last 22 amino acids (not binding to the plasma membrane). This cell line is transiently transfected with the degraders’ plasmid that contain a mCherry reporter (Mitochondrial Targeting Sequence, MTS). The degradation is analyzed by flow cytometry to determine the median fluorescence intensity (MFI) of the GFP in the transfected cells (mCherry positive cells). (B) Validation of sdAb ALFA/ALFA tag couple as functional degrader. HeLa S3/H2B-GFP-ALFA-KRAS^G12V^_166_ cells are transfected with sdAb ALFA or sdAb Ctl fused to three commonly used E3 ubiquitin ligases (VHL, SPOP, UBOX). GFP MFI is quantified by flow cytometry in the mCherry positive cells and normalized to the GFP MFI of the negative control. (C) Screening of 7 selected E3 ubiquitin ligases fused to sdAb ALFA. HeLa S3 H2B-GFP-ALFA-KRAS^G12V^_166_ cells are transfected with a negative control (VHL-sdAb Ctl), a positive control (VHL-sdAb ALFA) and 7 E3 ligases fused to sdAb ALFA. GFP MFI is quantified by flow cytometry and normalized as in (B). (D) HeLa S3 H2B-GFP-ALFA-KRAS^G12V^_166_ cells are transfected with the indicated constructs and treated (+) or not (−) with 0.8 μM of the proteasome inhibitor epoxomicin for 20 h. GFP MFI is quantified by flow cytometry and normalized as in (B). Statistical significance was determined by unpaired Student’s t test test in (B and D) and one-way ANOVA followed by Dunnett post-hoc tests in (C) (∗∗*p* < 0.01; ∗∗∗*p* < 0.001; ∗∗∗∗*p* < 0.0001; ns, not significant). Error bars in (B–D) correspond to mean values ± standard deviation (SD) of three independent biological repeats. See also Figures S1 and S2.

To identify E3 ligases suitable for TPD, we further designed a chromatin-anchored POI, by adding histone H2B on the chimeric POI GFP-ALFA-KRAS^G12V^_166_. We then stably expressed H2B-GFP-ALFA-KRAS^G12V^_166_ in HeLa S3 cell line (Figure 1A, left panel). We first tested whether sdAb ALFA fused to E3 ligases known for TPD (VHL,^28^ SPOP^27^ and the UBOX domain of the carboxyl-terminus of Hsc70-interacting protein [CHIP] ligase^30^) could mediate the degradation of this POI expressed in HeLa S3 cells. We modified our bicistronic degrader vector^5^ to express on one hand an FLAG-tagged sdAb ALFA-E3 ligase fusion and on the other hand, a mCherry targeted to the mitochondria as fluorescent reporter of transfection (Figure 1A, middle panel). 48 h after transiently transfecting the degrader constructs in these HeLa S3 stable cells, we showed by flow cytometry analysis (Figure 1A, right panel; Figure S2A) that all three E3 ligases significantly decreased by more than 75% the GFP signal of H2B-GFP-ALFA-KRAS^G12V^_166_ compared to their respective negative control (Figures 1B and S2B). These data demonstrate that the chromatin-anchored POI can be efficiently degraded by different sdAb ALFA-E3 ligase constructs. Hence, we decided to use this POI as model system to test novel E3 ligases amenable to TPD.

### Identification of an E3 ligase amenable to biodegrader

Only a minority of the over 600 E3 ligases in the human genome has been harnessed for TPD, suggesting that the potential of UPS has yet to be fully explored. To identify alternative E3 ligases amenable to TPD with intracellular protein binders, we considered several criteria. We first selected seven E3 ligases from different families, including members from the large and well-studied Cullin RING Ligase (CRLs) family (Table 1). CRLs function as multi-subunit complexes with a cullin serving as a scaffold that bridges an E2 enzyme to the substrate.^31^ Generally, an adaptor protein, containing a domain that interacts with different substrate receptors, links the substrate to the complex at the amino-terminal end of the cullin.^31^ We chose one representative E3 ligase of the six main CRLs subfamilies (CUL1-5 and CUL7) excluding CUL4, as many E3s from this subfamily have been proven unsuitable for TPD with biodegraders.^11^^,^^27^^,^^32^ In addition, we included CDC20, from the multi-subunit APC/C RING E3 ligase, and RNF144B from the RING between RING (RBR) family. We also considered in this selection process the natural subcellular localization and tissue expression of E3 ligases using the UbiHub online resource.^33^ We preferred E3 ligases that are reported to be expressed without any specific subcellular localization and, if possible, with ubiquitous expression across tissues. Next, we replaced the natural substrate recognition motif of each E3 ligase receptor with the sdAb ALFA protein binder (Table 1). Of note, RNF144B has a characteristic structure with two RING domains separated by an in-between RING (IBR),^34^ thus we fused the protein binder to either its amino- or its carboxy-terminal end. These chimeric constructs were then inserted in the bicistronic degrader vector and transiently transfected into HeLa S3/H2B-GFP-ALFA-KRAS^G12V^_166_ stable cell line. Flow cytometry analysis of GFP intensity revealed that among the seven E3 ligases tested, only the sdAb ALFA-SOCS7 construct showed a significant decrease in GFP intensity comparable to the positive control VHL-sdAb ALFA (≈90% of GFP signal depletion, Figure 1C). RNF144B-sdAb ALFA and FBX38-sdAb ALFA degraders exhibited moderate activity, significantly reducing GFP intensity by 50% (Figure 1C). Interestingly, the sdAb ALFA-RNF144B degrader displayed reduced potency compared to RNF144B-sdAb ALFA (25% versus 50% reduction, respectively), confirming that positioning of the E3 ligase on the sdAb is an important parameter to consider for an efficient TPD. However, FBXW8-, CDC20-, FEM1B-, and KLHL7 E3 ligases-sdAb ALFA fusions did not decrease the GFP intensity and therefore were not active in these conditions. Importantly, all degrader constructs were confirmed to be expressed by western blotting (Figure S2C). We further investigated the subcellular localization of KLHL7, RNF144B, SOCS7, and the three control E3 ligases (VHL, UBOX, and SPOP) fused to a 3xFLAG tag by immunofluorescence in HeLa S3 cells (Figure S2D). We showed that KLHL7, which was not active in the screening (Figure 1C), had mostly a cytoplasmic localization (Figure S2D). Nevertheless, as per our E3 ligase selection process, SOCS7 and to a lesser extent RNF144B, had a diffuse distribution throughout the cells (including both the nucleus and cytoplasm) compared to the known nuclear-localized SPOP^27^ (Figure S2D).

**Table 1:** Description of the E3 ligases used in the protein-based degrader screening

E3 ligase	E3 complex/family^a^	Region used^b^	Size (kDa)^c^	Positioning on the sdAb^d^
FBX38	SKP1-Cul1-F-box	1-75 (/1188 aa)	8.8	Amino-terminus
FBXW8	FBXW8-SKP1-Cul7-RBX1	1-197 (/598 aa)	22.6	Amino-terminus
KLHL7	BTB-Cul3-RBX1	1-250 (/586 aa)	28.4	Amino-terminus
CDC20	Anaphase promoting complex/cyclosome (APC/C)	1-181 (/499 aa)	19.8	Amino-terminus
FEM1B	FEM1B-EloB-EloC-Cul2-RBX1	327-627 (/627 aa)	34.1	Carboxy-terminus
SOCS7	SOCS7-EloB-EloC-Cul5-RBX2	501-581 (/581 aa)	9.7	Amino- and carboxy-terminus
RNF144B	RBR	1-258 (/303 aa)	29	Amino- and carboxy-terminus
VHL	VHL-EloB-EloC-Cul2-RBX1	1-213 (/213 aa)	24.1	Amino-terminus
SPOP	SPOP-Cul3-RBX1	167-374 (/374 aa)	23.1	Carboxy-terminus
CHIP	UBOX	128-303 (UBOX) (/303 aa)	20.9	Carboxy-terminus

Truncations have been designed to replace the original substrate binding domain by the sdAb (except for VHL, full size protein).

a E3 complex/family: E3 ubiquitin ligase complex or family they function in.

b Region used: domain of the E3 ligase fused to the sdAb (/aa: total number of amino acids corresponding to the full-length E3 ligase).

c Size: molecular weight in kDa of the selected region.

d Positioning on the sdAb: position of the E3 ligase domain in the fusion with the sdAb.

Finally, to demonstrate that the decreased GFP intensity induced by SOCS7-based biodegrader is mediated through proteasomal degradation, cells expressing sdAb ALFA-SOCS7 or sdAb Ctl-SOCS7 were treated with epoxomicin, a proteasome inhibitor.^35^ While the GFP intensity significantly decreased in untreated cells expressing sdAb ALFA-SOCS7 compared to the negative control, the GFP intensity was restored in epoxomicin-treated cells expressing sdAb ALFA-SOCS7 (Figure 1D). This result demonstrates that SOCS7-based biodegrader degrades selectively H2B-GFP-ALFA-KRAS^G12V^_166_ in a proteasome dependent manner. Hence, we chose to further characterize SOCS7 and compare its performance with that of the commonly used VHL/SPOP/UBOX E3 ligases.

### SOCS7 is a versatile E3 ligase for TPD

**Figure 2:**
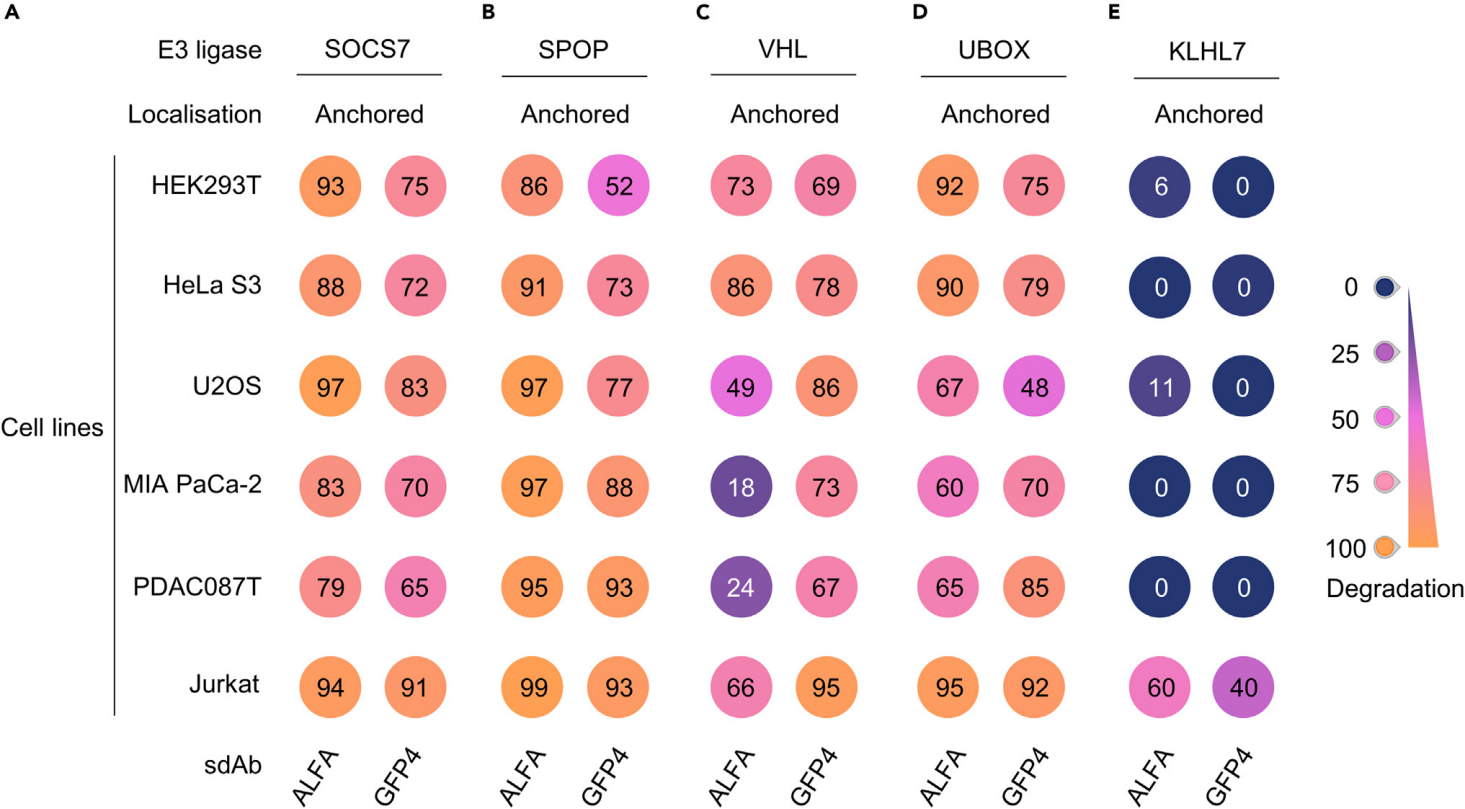
SOCS7-based biodegraders potently deplete a chromatin-anchored POI Representation of the percentage of degradation (normalized GFP MFI) in colored circles (no degradation in dark blue, 50% in pink, to 100% of degradation in orange). Six different cell lines used from different tissues: HeLa S3 (cervix), HEK293T (embryonic kidney), U2OS (bone), MIA PaCa-2 (pancreas), PDAC087T (pancreas, patient-derived) and Jurkat (blood). (A) SOSC7 is either fused to sdAb ALFA or sdAb GFP4 and tested in the cell lines expressing the chromatin-anchored POI H2B-GFP-ALFA-KRAS^G12V^_166_. (B–D) SPOP (B), VHL (C), UBOX (D) and KLHL7 E3 ligase (E) that did not show degradation in the screening assay, are tested in the same conditions as in panel (A). Each experiment in (A–E) was performed at least three times (independent biological repeats). See also Figures S3 and S4.

Previous studies have shown that some E3 ligases can efficiently degrade proteins in a restricted set of cell lines, thereby limiting their applicability for TPD.^21^^,^^27^ Therefore, when evaluating a new E3 ligase for TPD, it is important to determine its capacity to degrade the same protein across multiple cell lines. Consequently, we studied the ability of SOCS7-based biodegraders to deplete H2B-GFP-ALFA-KRAS^G12V^_166_ across various cell lines. To this end, we systematically compared the degradation efficiency of sdAb ALFA-SOCS7 and sdAb Ctl-SOCS7 in six cell lines from different origins. We used HEK293T (embryonic kidney cells), HeLa S3 (cervix cancer cells), U2OS (osteosarcoma cells), MIA PaCa-2 (pancreatic cancer cells), PDAC087T (patient-derived pancreatic cancer cells), and Jurkat cells (T cell acute lymphoblastic leukemia cells) that stably expressed the H2B-GFP-ALFA-KRAS^G12V^_166_ construct (Figure S3A). 48 h after transient transfection of the degraders, the intensity of GFP was analyzed by flow cytometry. We showed that sdAb ALFA-SOCS7 decreased the GFP intensity by more than 75% across all cell lines tested (Figure 2A, left panel; Figure S3B). We also assessed sdAb ALFA and its negative control fused to VHL, UBOX, and SPOP well-known E3 ligases and KLHL7 identified as negative in the screening (Figure 1C). VHL, UBOX, and SPOP-based biodegraders depleted the GFP signal in all cell types but with different efficiencies (Figures 2B–2D, left panel; Figures S3C–S3E). SPOP-based biodegrader showed similar efficacy to SOCS7-based biodegrader, with more than 75% of GFP signal reduction (Figures 2A, 2B, S3B, and S3C). These two biodegraders displayed stronger activity than VHL and UBOX fused to sdAb ALFA across all cell lines tested (Figures 2A–2D and S3B–S3E). To determine whether the difference of activity displayed by the biodegraders was related to their expression level, we assessed the expression of sdAb ALFA-E3 ligase degraders by western blotting. Our results indicate that all biodegraders were expressed, except for VHL-sdAb ALFA, which showed a weak, to undetectable, expression in cells (Figures S4A–S4E). On the contrary, high expression levels were detected for SPOP- and UBOX-based biodegraders (Figures S4A–S4E), suggesting that the weaker activity of the UBOX-based biodegrader is not linked to its expression level. However, the low level of expression of VHL-sdAb ALFA might explain why it was less efficacious in pancreatic cancer cells, with 25% of GFP signal drop in MIA PaCa-2 and PDAC087T, compared to above 50% in the other cell lines (Figure 2C, left panel; Figure S3D). Finally, KLHL7-sdAb ALFA could significantly diminish the GFP signal in Jurkat cells, while it was unable to degrade H2B-GFP-ALFA-KRAS^G12V^_166_ in the other cells (Figures 2E and S3F), including HeLa S3 that was used for the initial E3 selection (Figure 1C). These data highlight the importance of testing various cell lines to fully capture the degradation landscape of a particular E3 ligase.

We and others have previously shown that changing the protein binder of a biodegrader can modify its ability to deplete its target.^11^^,^^32^^,^^36^ To further characterize the versatility of SOCS7-mediated degradation, we replaced the protein binder in SOCS7-based biodegrader with an anti-GFP sdAb, sdAb GFP4.^37^ This was also carried out on the other E3 ligase-based biodegraders. Flow cytometry analysis showed that sdAb GFP4-SOCS7, as well as sdAb GFP4-VHL/SPOP/UBOX fusions, decreased GFP intensity in all cell lines (Figures 2A–2E, right panel). Remarkably, sdAb GFP4-KLHL7 retained its ability to efficiently reduce the GFP signal in Jurkat cells compared to sdAb ALFA-KLHL7 (Figure 2E, right panel; Figure S3F). Western blot analysis of sdAb GFP4-E3 ligase degraders revealed a pattern of expression similar to that observed for sdAb ALFA-E3 ligase degraders (Figures S4A–S4E). In conclusion, these data suggest that substituting the protein binder component in SOCS7-based biodegrader does not modify its activity.

**Figure 3:**
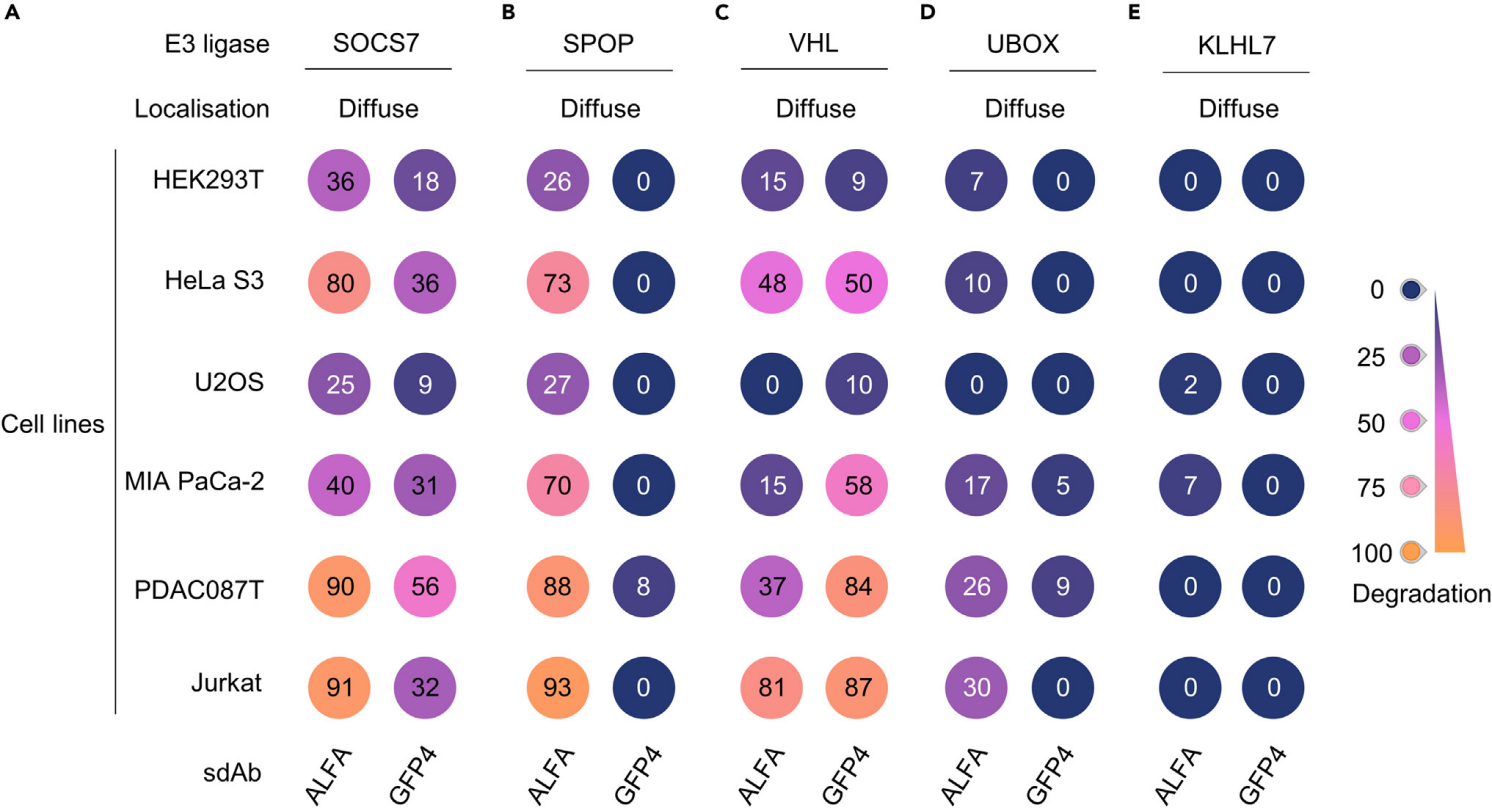
SOCS7-based biodegraders degrade a diffuse POI Representation of the percentage of degradation (normalized GFP MFI) in colored circles (no degradation in dark blue, 50% in pink, to 100% of degradation in orange). (A) SOSC7 is either fused to sdAb ALFA or sdAb GFP4 and tested in the indicated cell lines expressing the diffuse POI GFP-ALFA-KRAS^G12V^_166_. (B–D) SPOP (B), VHL (C), UBOX (D) and KLHL7 E3 ligase (E) that did not show degradation in the screening assay, are tested in the same conditions as in panel (A). Each experiment in (A–E) was performed three times (independent biological repeats). See also Figures S5 and S6.

Several reports have shown that the ability of some E3 ligase-based degraders to efficiently degrade a protein depends on its subcellular localization.^21^^,^^26^ Therefore, we stably expressed GFP-ALFA-KRAS^G12V^_166_ (i.e., a diffuse protein) in the same cell lines. Compared to the chromatin-anchored POI, it displayed a diffuse pattern in cells (Figures S3A and S5A). After transient expression of sdAb ALFA- or sdAb GFP4-E3 ligase constructs in the aforementioned cell lines, we analyzed the intensity of GFP by flow cytometry. Globally, the degradation activity declined for all biodegraders (Figures 3A–3E). This difference was more important for UBOX-based biodegraders that barely depleted the GFP signal in this condition with no more than 30% reduction of the GFP signal (Figures 3D and S5E). In contrast to the chromatin-anchored construct, KLHL7-based biodegraders failed to decrease the GFP intensity of the diffuse POI in Jurkat cells (Figures 3E and S5F). Surprisingly, while sdAb ALFA-SPOP degraded GFP-ALFA-KRAS^G12V^_166_ construct (Figure 3B, left panel; Figure S5C), the sdAb GFP4-SPOP fusion did not (Figure 3B, right panel; Figure S5C), highlighting the limitation of sdAb GFP4-SPOP degrader. However, SOCS7 was the only E3 ligase able to induce the degradation of GFP-ALFA-KRAS^G12V^_166_ across all cell lines with both sdAb ALFA and GFP4 (Figures 3A and S5B). Importantly, in GFP-ALFA-KRAS^G12V^_166_ cell lines, all biodegrader constructs had a similar pattern of expression that the one found in H2B-GFP-ALFA-KRAS^G12V^_166_ cell lines (Figures S6A–S6E).

Altogether, these data underline that SOCS7 is a robust and versatile E3 ligase amenable to TPD with efficacy maintained independently of the cell type, target intracellular localization and target binder.

### The geometry of SOCS7-based biodegraders impact their activity

**Figure 4:**
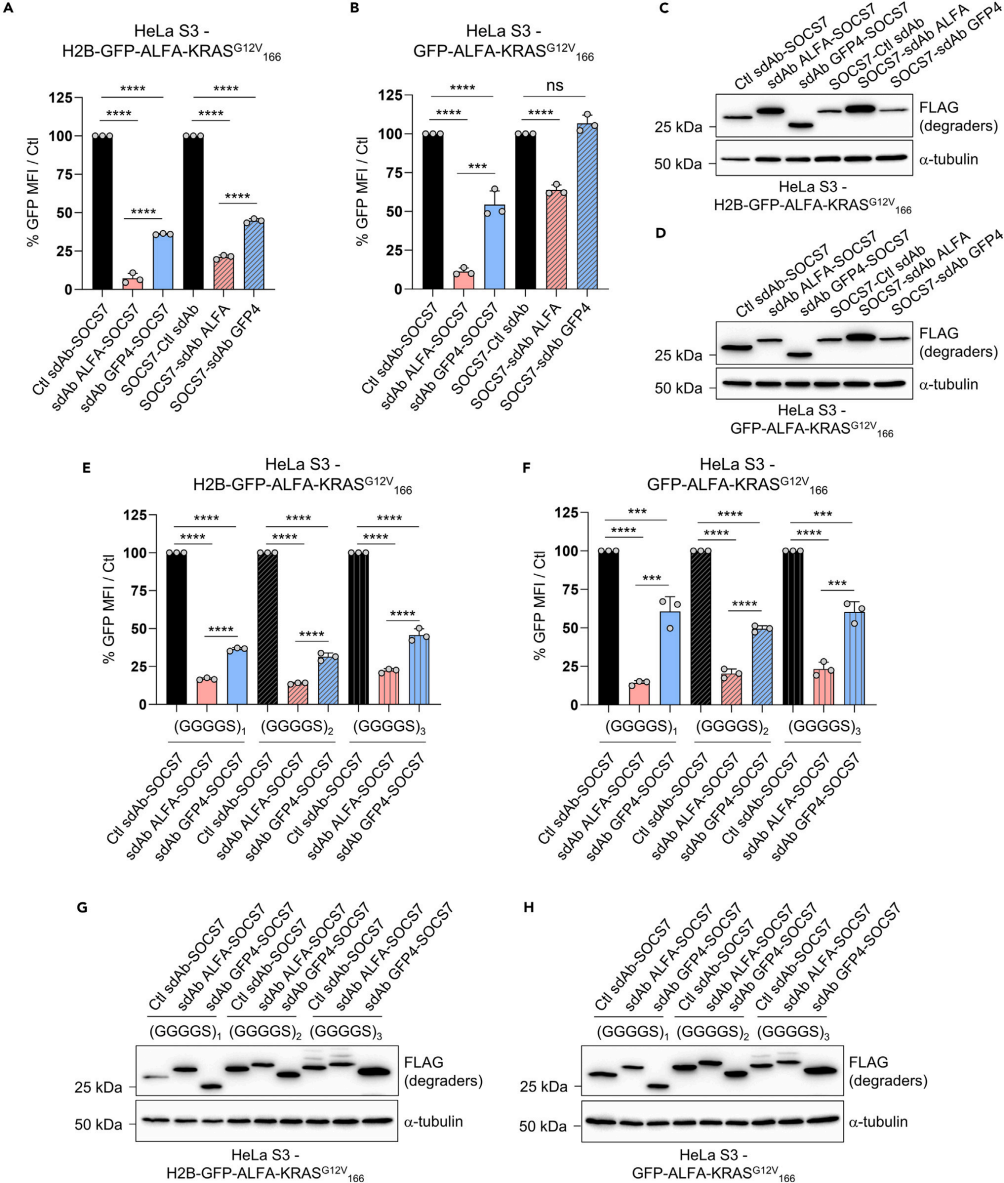
The geometry of SOCS7-based biodegraders impact their activity (A and B) SOCS7 fused to either the amino- or the carboxy-terminal end of sdAb Ctl, ALFA or GFP4 is transfected in HeLa S3 H2B-GFP-ALFA-KRAS^G12V^_166_ (A) or HeLa S3 GFP-ALFA-KRAS^G12V^_166_ (B). GFP MFI in transfected cells is determined by flow cytometry and normalized to Ctl GFP MFI. (C and D) The control of expression of SOCS7-based biodegraders used in panel A (H2B-GFP-ALFA-KRAS^G12V^_166_, [C]) and panel B (GFP-ALFA-KRAS^G12V^_166_, [D]) was assessed by western blot. α-tubulin is the loading control. (E and F) The linker length of SOCS7-based biodegraders (Ctl, ALFA or GFP4) was increased from (GGGGS)_1_ to (GGGGS)_2_ and to (GGGGS)_3_. These constructs were transfected in HeLa S3 H2B-GFP-ALFA-KRAS^G12V^_166_ (E) or HeLa S3 GFP-ALFA-KRAS^G12V^_166_ (F), GFP MFI in transfected cells is determined by flow cytometry and normalized to Ctl GFP MFI. (G and H) The control of expression of the biodegraders used in panels E and F was analyzed by western blot. α-tubulin is the loading control. Statistical significance in panels A, B, E, and F was determined by one-way ANOVA followed by Tukey post-hoc tests (∗∗∗*p* < 0.001; ∗∗∗∗*p* < 0.0001; ns, not significant). Error bars in (A, B, E, and F) are mean ± SD of three independent biological repeats. A representative experiment out of two independent biological repeats is shown in (C, D, G, and H).

Modifying the geometry of a degrader, e.g., the positioning of the E3 ligase and the linker length between the sdAbs and the E3 ligase, may substantially change the activity of the corresponding degrader. We first assessed the influence of the relative positioning of SOCS7 and sdAbs in the biodegraders on their ability to deplete their target. We showed that fusing SOCS7 to the amino-terminus of sdAb ALFA still enabled a significant degradation of both anchored and diffuse POIs expressed in HeLa S3 (≈75% and 50% reduction in GFP signal, respectively) in HeLa S3 but with lower efficacy compared to the carboxy-terminus (≈90% drop of GFP intensity, Figures 4A and 4B). Notably, when SOCS7 was fused to the amino-terminal position of sdAb GFP4, it did not deplete GFP-ALFA-KRAS^G12V^_166_ anymore (Figure 4B). Importantly, modifying the position of SOCS7 on the sdAbs did not alter the expression level of the corresponding biodegraders (Figures 4C and 4D).

While the linker length is another critical parameter for the activity of a chemical-based degraders,^2^ this parameter is rarely assessed for biodegraders. Therefore, we tested whether modifying the linker length between the sdAbs and SOCS7 could alter the degradation potency of the corresponding biodegrader. We inserted one, two, or three repetitions of a four glycine/one serine motif (GGGGS)_1-3_ between sdAbs and SOCS7 and transfected these constructs in HeLa S3 H2B-GFP-ALFA-KRAS^G12V^_166_ and GFP-ALFA-KRAS^G12V^_166_ (Figures 4E–4H). The GFP intensity analysis by flow cytometry showed that the linker length had minimal impact on SOCS7-based biodegraders activity (Figures 4E and 4F). Furthermore, western blot analyses showed that the linker length had no effect on the expression level of the corresponding biodegraders (Figures 4G and 4H). These results indicate that the efficacy of the tested SOCS7-based biodegraders is primarily driven by the relative positioning of SOCS7 and sdAbs, highlighting the importance of the design of the fusion of sdAbs with the E3 ligase domain to achieve optimal degradation.

### The affinity of sdAb ALFA modifies the activity of SOCS7-based sdAb ALFA degrader

**Figure 5:**
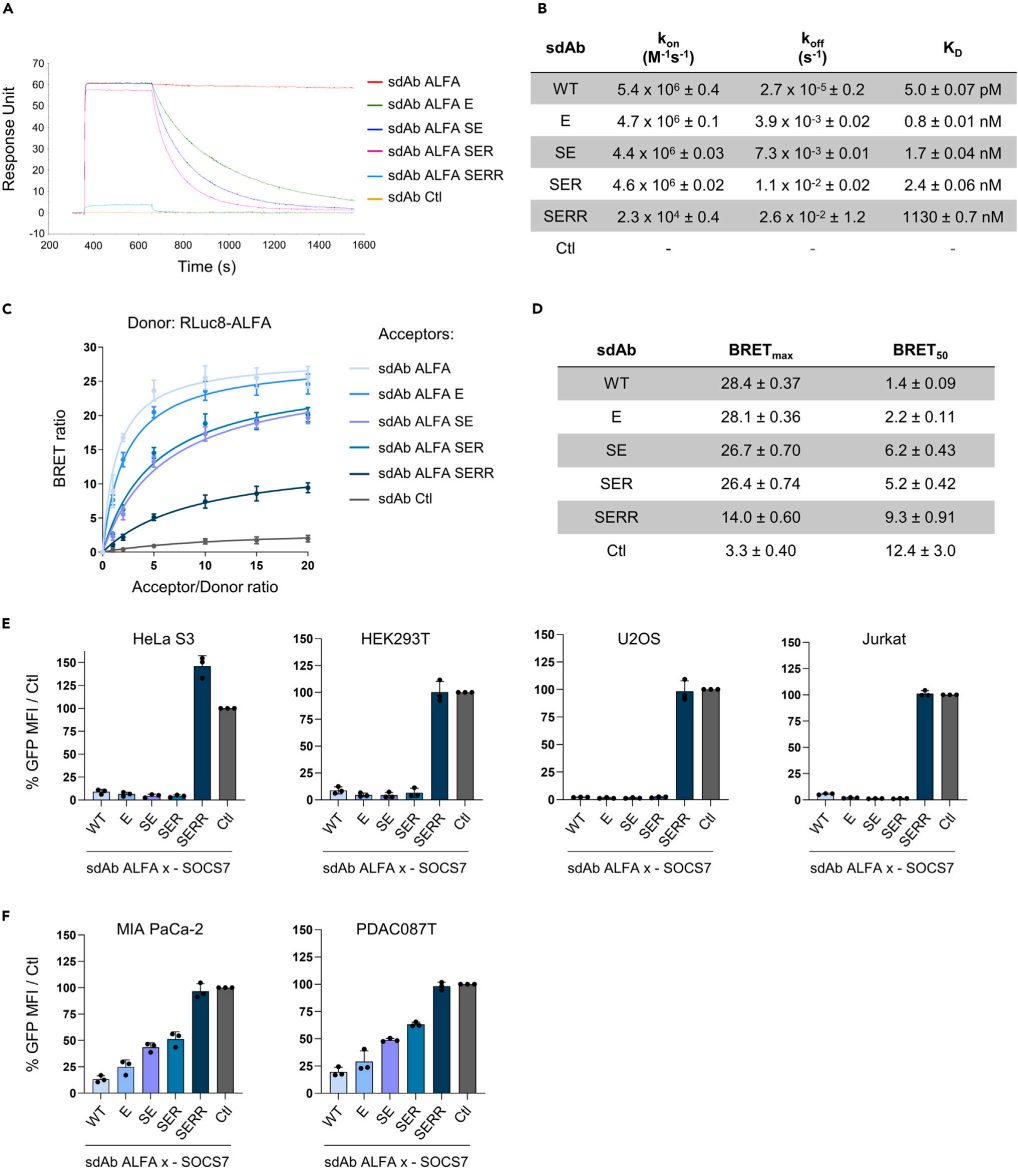
The affinity of sdAb ALFA in SOCS7-based biodegrader impacts its efficacy of degradation (A) Representative Surface Plasmon Resonance (SPR) sensorgram for sdAb ALFA and its mutants (at 50 nM) to an ALFA-tagged human Fc protein. (B) Summary of affinity data obtained by SPR from two technical repeats: k_on_, k_off_ and K_D_ mean ± SD. (C) BRET donor saturation assay between ALFA-tag (donor) and the acceptors sdAbs ALFA WT and its mutants. (D) BRET_max_ and BRET_50_ values ±SD from the donor saturation curves displayed in (C). (E) Normalized GFP MFI quantified by flow cytometry of H2B-GFP-ALFA-KRAS^G12V^_166_ HeLa S3, HEK293T, U2OS and Jurkat cells transfected with sdAb Ctl and the different sdAb ALFA mutants fused to SOCS7. (F) Normalized GFP MFI from pancreatic cell lines MIA PaCa-2 and PDAC087T expressing H2B-GFP-ALFA-KRAS^G12V^_166_ and transfected with the same sdAbs as in (E). Error bars in (C, E, and F) are mean ± SD of three independent biological repeats. See also Figures S7 and S8.

The degradation efficiency of small molecule-based PROTACs is not dependent on the affinity of the substrate binder.^38^ However, little is known on how the affinity of a protein binder impacts the degradation efficiency of its corresponding biodegrader. Therefore, we assessed whether the affinity of sdAb ALFA modifies its capacity to degrade its target when fused to SOCS7. We designed four variants of sdAb ALFA, where amino acids involved in ALFA binding were incrementally mutated, generating mutants called sdAb ALFA E, SE, SER, SERR that correspond to the mutated amino acids on the sdAb sequence (Figure S1A). We then produced and purified these four mutants together with sdAb Ctl and the parental sdAb ALFA to determine their affinity for ALFA-Fc by surface plasmon resonance (SPR) (Figures 5A and S7A). SPR data confirmed that sdAb Ctl did not bind to the ALFA tag while the parental sdAb showed a picomolar affinity as originally reported^29^ (Figures 5A, 5B, and S7A). sdAb ALFA E, SE and SER mutants had K_D_ within the low nanomolar range while the K_D_ of the SERR mutant was within the micromolar range (Figure 5B). We next validated these data in a cell-based approach using a BRET donor saturation assay in HEK293T cells (Figure 5C). Mutations in the parental sdAb ALFA reduced their corresponding BRET signal (Figure 5C) without affecting their expression level as demonstrated by similar GFP^2^ signal between all sdAbs (Figure S8A). This signal difference was quantified by an increased BRET_50_ value (a proxy of the relative affinity of the sdAb ALFA mutants to ALFA tag^39^) and a decreased BRET_max_ value, together consistent with a reduced affinity of the mutants toward the ALFA tag (Figure 5D). Interestingly, the BRET_50_ values globally ranked the mutants following their K_D_, further demonstrating the utility of this method to assess relative affinities within the cell.^40^ Finally, we controlled whether the mutations introduced in the sdAb ALFA could affect the folding properties of the different mutants by using the nano differential scanning fluorimetry (nanoDSF) method. All sdAbs showed a T_onset_ (the temperature at which the protein starts to unfold) higher than 55°C (Figure S7B), significantly exceeding the temperatures used in SPR and cellular assays (room temperature and 37°C, respectively). Remarkably, sdAb SERR and Ctl (with four and five mutations in comparison to the parental sdAb ALFA, respectively) displayed the highest thermal stability compared to the other mutants and parental sdAb ALFA. In addition, the temperatures of aggregation (T_agg_) for these two variants were higher than 90°C, underscoring their inability to undergo aggregation (Figure S7B). Overall, these data rule out the possibility that improper folding, low stability, or aggregation are the underlying causes of the reduced affinity of the sdAb mutants.

Next, we cloned these mutants fused to SOCS7 in the bicistronic degrader vector and transiently transfected them into the six cell lines stably expressing the H2B-GFP-ALFA-KRAS^G12V^_166_ construct. The degradation pattern shown by the mutants was dependent on the cell line. In HeLa S3, HEK293T, U2OS, and Jurkat cells, biodegraders made of sdAb mutants with a K_D_ in the nanomolar range were as efficient as the parental sdAb ALFA-SOCS7 degrader (Figure 5E). However, in pancreatic cancer cells (MIA PaCa-2, PDAC087T), the degradation efficiency of the sdAb mutants-SOCS7 was linked to the affinity of the corresponding mutated sdAbs (Figure 5F). Interestingly, sdAb ALFA SERR-SOCS7, which has a micromolar K_D_, was ineffective in all tested cell lines, showing similar GFP intensity to the negative control (Figures 5E and 5F). We then investigated whether differences in the expression of components of the SOCS7 E3 ligase complex, including the biodegraders, could explain the discrepancies between pancreatic cancer cells and the other cell lines. We showed by western blot experiments that all biodegraders were expressed at similar levels in HeLa S3, HEK293T, and MIA PaCa-2 (Figures S8B–S8D), thus excluding a difference of expression between mutants. Variation of expression of other proteins forming SOCS7 E3 ligase protein complex may explain the difference of degradation between cell lines. We next confirmed that CUL5, Elongin B/C, and RBX2, essential partners required for SOCS7 to form a functional E3 ligase complex, were expressed in the six cell lines (Figure S8E), thus ruling out a lack of expression as a possible explanation. Finally, we tested further whether the behavior of the biodegraders would be altered when changing the localization of the POI. We transiently transfected the mutants and parental sdAb ALFA-SOCS7 biodegrader constructs in HeLa S3, HEK293T, MIA PaCa-2, and PDAC087T stably expressing GFP-ALFA-KRAS^G12V^_166_ and quantified the GFP MFI of the transfected cells by flow cytometry (Figure S8F). While both sdAb Ctl and sdAb ALFA SERR-based degraders were inactive in all tested cell lines (Figure S8F), the four other biodegraders equally decreased the GFP intensity in HeLa S3 and HEK293T/GFP-ALFA-KRAS^G12V^_166_, as well as in the pancreatic cancer cell lines MIA PaCa-2 and PDAC087T/GFP-ALFA-KRAS^G12V^_166_ (Figure S8F).

Altogether, these results suggest that unlike chemical PROTACs, the affinity of the protein binder forming a biodegrader is a critical parameter to consider for efficient TPD. Indeed, no degradation was observed above an affinity in the micromolar range with the sdAb ALFA-SOCS7 system. Importantly, the high affinity parental sdAb ALFA resulted in the best degradation capacity when combined to SOCS7 irrespective of the cell lines and POI, suggesting the importance of optimizing the affinity of the protein binder.

### Degradation of endogenous KRAS protein with SOCS7-based KRAS biodegrader

**Figure 6:**
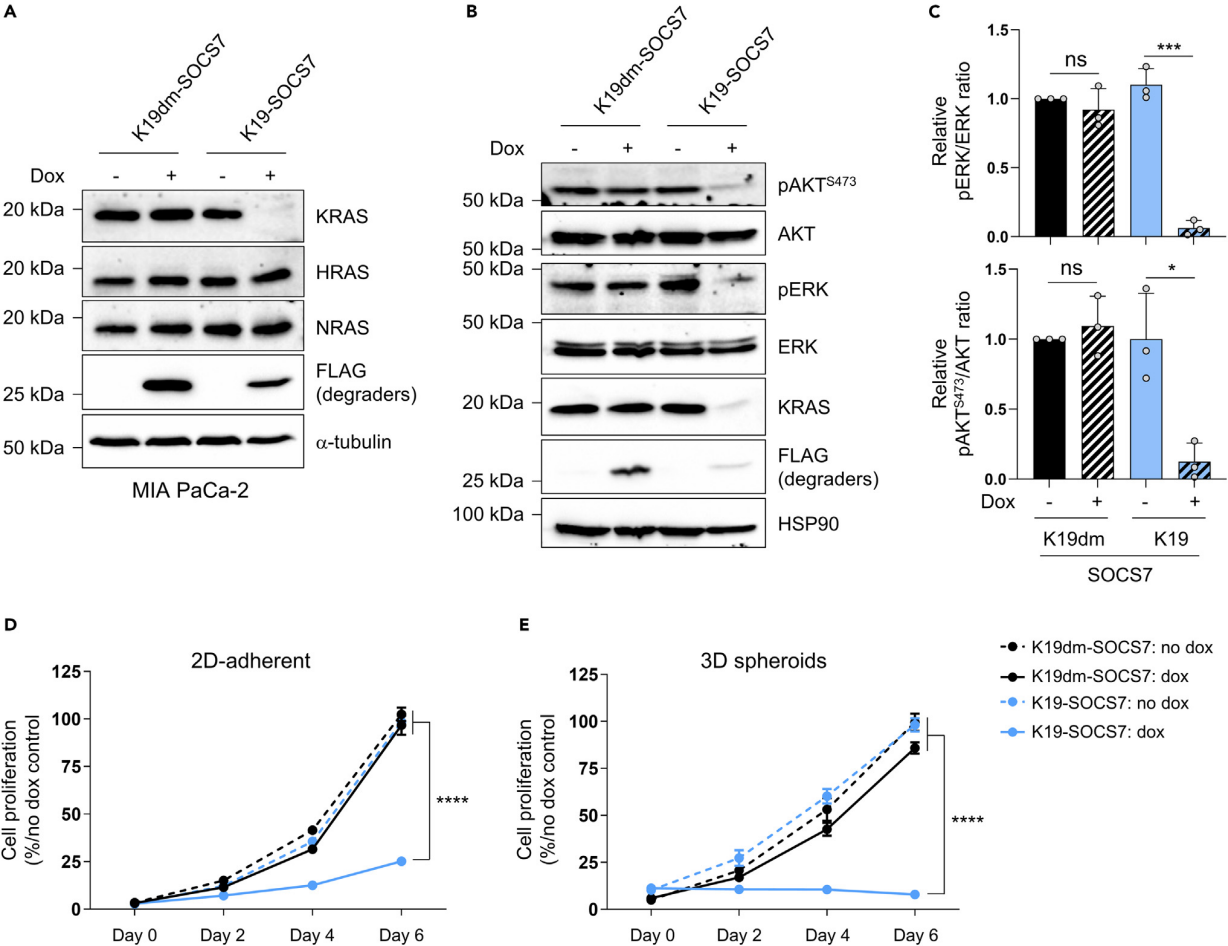
SOCS7-based KRAS degrader inhibits pancreatic cancer cells proliferation (A) Efficacy of SOCS7-based KRAS degrader to deplete its target in MIA PaCa-2 compared to a negative degrader (K19dm-SOCS7). KRAS, HRAS and NRAS protein levels were analyzed by western blot after 72 h of dox treatment of the cells at 0.5 μg mL^−1^ (+) or not induced (−). Expression of the degraders is shown with the FLAG antibody. α-tubulin is the loading control. (B) Effect of SOCS7-based KRAS degrader on RAS downstream signaling pathways of MIA PaCa-2 analyzed by western blot. HSP90 is the loading control. Cells were induced with dox with the same conditions indicated in (A). (C) Quantification of pAKT^S473^/AKT and pERK/ERK signals from (B). The signals were normalized to the no dox condition of K19dm-SOCS7. (D and E) Assessment of the effect of SOCS7-based KRAS degrader on 2D-adherent (D) and 3D spheroids (E) proliferation of MIA PaCa-2 cells. These proliferation assays (2D and 3D) were normalized to the no dox condition for each cell line. The dotted lines represent the no dox condition and the plain lines show the dox-treated cells. Statistical analyses were performed using an unpaired t-test (C) or a one-way ANOVA followed by Tukey post-hoc tests (D and E) (∗*p* < 0.05; ∗∗∗*p* < 0.001; ∗∗∗∗*p* < 0.0001; ns, not significant). Each experiment was performed three (A–C) or four (D and E) times. Error bars in (C–E) are mean ± SD from at least three biological repeats. See also Figure S9.

All the data reported previously were obtained using a chimeric non-natural protein as target and SOCS7-based biodegraders made of anti-tag sdAbs (against the ALFA or GFP tags). To fully characterize the versatility of SOCS7, it is crucial to test whether this E3 ligase (i) functions with a protein scaffold different from sdAbs and (ii) enables the depletion of an endogenous protein. To do that, we used a previously reported KRAS-specific binder (i.e., not binding NRAS or HRAS), the designed ankyrin repeat protein (DARPin) K19 and its negative control K19dm (not binding to KRAS).^16^ We fused K19 and K19dm to SOCS7 and inserted them in the bicistronic degrader vector. We first compared the performance of this new biodegrader to degrade the chimeric H2B-GFP-ALFA-KRAS^G12V^_166_ in HeLa S3 cells with the sdAb ALFA-SOCS7. GFP intensity evaluated by flow cytometry showed that K19-SOCS7 reduced the GFP signal at least as well as sdAb ALFA-SOCS7 (Figure S9A). Next, we investigated whether K19-SOCS7 could enable the degradation of endogenous KRAS in MIA PaCa-2, a KRAS^G12C^ mutant pancreatic cancer cell line. To achieve this, we established stable MIA PaCa-2 cell lines that expressed either K19dm-SOCS7 or K19-SOCS7 under the control of doxycycline (dox). As expected, after dox induction of the degraders’ expression (detected by an FLAG tag, Figure 6A), K19-SOCS7 specifically depleted KRAS protein while sparing NRAS or HRAS (Figure 6A). We confirmed that the degradation was proteasome dependent by treating cells with epoxomicin. In western blotting experiments, cells treated with epoxomicin for 18 h failed to degrade KRAS upon dox-induced expression of K19-SOCS7 (Figure S9B). Next, we investigated the consequences of KRAS depletion on its downstream signaling pathways. We showed that KRAS degradation significantly decreased the phosphorylation of protein kinase B (AKT) and extracellular signal-regulated kinase (ERK) kinases indicating that it impeded the activation of Phosphatidylinositol 3-kinase (PI3K) and Mitogen-activated protein kinase (MAPK) pathways, respectively (Figures 6B and 6C). Finally, we tested the functional consequences of KRAS degradation on MIA PaCa-2 cells’ proliferation in 2D-adherent and 3D-spheroid settings. KRAS depletion by K19-SOCS7 significantly blocked 2D-adherent and 3D spheroids cell proliferation after 6 days of dox treatment compared to the negative control K19dm-SOCS7 (Figures 6D and 6E). Altogether, these results show that a SOCS7-based biodegrader can efficiently degrade an endogenous protein, further strengthening SOCS7 versatility for TPD.

## Discussion

One challenge in the field of TPD is the discovery of alternative E3 ligases that can be recruited. Indeed, only a few of these have been harnessed for TPD out of the more than 600 known E3 ligases. In this study, we identified three E3 ligases enabling TPD: RNF144B, FBX38, and SOCS7 through a protein-based degrader approach performed in HeLa S3 cells. Interestingly, two recent reports used a similar method to screen E3 ligases amenable to TPD with shared screened E3 ligases. Poirson et al. employed a protein-based degrader high-throughput screening assay using an ORFeome library to unveil functional E3 ligases for TPD.^21^ However, SOCS7, which was part of the ORFeome library, was not identified as a functional E3 for TPD in their study, while it was the most potent E3 ligase in our screening approach. Similarly, Röth et al. uncovered KLHL7 in a cell-based screening using a protein-based degrader approach,^25^ but it was not functional in our study. Interestingly, both screenings were performed in HEK293T cells, where we found that SOCS7 is active and KLHL7 not active as biodegrader, suggesting that the cell type might not account for the differences in activity observed between these studies and ours. A second parameter that might contribute to the discrepancies between these studies and ours is the design of the E3 ligase-based biodegraders. Poirson and Röth studies used full-length SOCS7 and KLHL7, respectively,^21^^,^^25^ while we made truncated version of these E3 ligases and observed opposite behavior for SOCS7 and KLHL7 compared to these other studies (i.e., active and inactive, respectively). More precisely, we removed the substrate-binding domain of each tested E3 ligase, in order to retain the minimal ligase domain required for its activity (and decrease the molecular weight of the biodegrader). However, such truncations may result in inactive or suboptimal E3 ligases and further optimization of the truncation might be requested to improve the biodegraders activity. Another key parameter is the position of the E3 ligase domain on the sdAb (either on the amino- or the carboxy-terminal end of the sdAb) that might impact the degradation efficacy of these biodegraders. Actually, to construct biodegraders, we kept the position of the natural substrate recognition motif of the E3 and replaced it with the protein binder. Indeed, we have previously demonstrated that not respecting this rule could negatively affect the degradation of RAS and KRAS with either UBOX or VHL.^8^ Nevertheless, we showed that modifying the position of RNF144B and SOCS7 E3 ligases on the protein binder could still lead to the target degradation, highlighting the versatility property of these E3 ligases. Finally, while the influence of the linker length and/or composition is well-described for chemical-based degraders,^2^ these parameters have not been explored for biodegraders thus far. Our data show a limited impact of the linker length between the sdAbs and SOCS7 on the activity of the corresponding biodegraders. This warrants further investigation to delve deeper into the influence of the linker on the activity of biodegraders. For instance, exploring different E3 ligase domains and degrader geometries or varying the amino acid composition of the linker could provide valuable insights. Altogether, these data indicate the difficulty to predict the right parameters to find functional E3 ligases for TPD, especially for high-throughput screenings.

While the search for disease-relevant or tissue-specific E3s is an ongoing research topic,^41^ identifying an E3 ligase that functions universally for TPD is crucial for the rapid development of effective biodegraders. An optimal E3 ligase should confer to a degrader the capacity of depleting its target regardless of the cell type, protein binder or POI nature, and localization. However, E3 ligases have shown variable degradation efficacy across different cell types. Shin et al. demonstrated that the F-BOX domain of Slmb E3 ligase from Drosophila melanogaster can promote H2B-GFP depletion in HeLa S3 cells but not in HEK293 cells.^27^ In addition, some E3 ligases do not efficiently degrade a target depending on its subcellular localization,^21^^,^^26^ while others are sensitive to the choice of protein binder. We have previously shown that various anti-GFP sdAbs, able to bind intracellular GFP, failed to mediate H2B-GFP degradation when fused to the F-BOX domain of Slmb,^36^ possibly due to differences in lysine accessibility on the POI. Indeed, different binders of the same POI do not necessarily share the same binding epitope nor the same binding’s orientation. Consequently, their lysine accessibility for the ubiquitin transfer may vary depending on these properties, ultimately modifying their potency as biodegraders.

To address these limitations, we investigated the versatility of SOCS7 as an E3 ligase for TPD. Our findings demonstrated that SOCS7, implemented as biodegrader, effectively degraded the POI in six distinct cell lines, expressing either a chromatin-anchored or diffuse POI, and was compatible with different types of protein binders (anti-ALFA or GFP sdAbs, anti-KRAS DARPin). Notably, SOCS7 outperformed the most commonly used E3 ligases VHL and SPOP under the tested conditions. In addition, a SOCS7-based biodegrader enabled the degradation of endogenous KRAS oncoprotein in pancreatic cancer cells, ultimately leading to the cells’ proliferation inhibition. These data further demonstrated the versatility of SOCS7 in depleting a POI from another subcellular compartment (i.e., a plasma membrane-bound POI) and its potential to study the functional consequences of a POI’s degradation in a relevant cellular model.

A comparison of the degradation activity of some E3 ligases across diverse cell lines highlighted different patterns of degradation. KLHL7-based biodegraders only degraded the anchored POI in Jurkat cells, a T cell line, while VHL depleted less efficiently the same anchored POI in pancreatic cancer cells. These results indicate the need to assess new E3 ligases against a panel of cell lines to ascertain their specificity and breadth of degradation.

Because little is known on the relationship between target binding affinity and degradation efficacy of biodegraders, we investigated the impact of the sdAb’s affinity on its degradation efficacy when fused to SOCS7. Unlike chemical-based degraders, for which a direct correlation between affinity and potency of degradation is challenging to establish,^38^^,^^42^ the ease to generate affinity variants of a protein binder without modifying the topology of the complex it forms with its target makes possible such studies for protein-based degraders. Lim et al. showed that only GFP binders with a K_D_ below 200 nM induced H2B-GFP degradation, suggesting that a threshold of affinity is required to deplete a target with protein binders.^11^ However, this result was obtained using distinct GFP binders that may have a different access to lysine residues and/or binding orientations, which could modify their intrinsic degradation ability. In another study, Teng et al. reported that modulating the affinity of the same KRAS binder by decreasing the number of lysine residues on its surface could modify its degradation efficacy without any direct correlation between affinity and degradation potency.^9^ Here, we generated sdAb ALFA mutants with a wide range of affinity (pM to μM range) without introducing or deleting lysine residues. We showed that the least affine sdAb ALFA mutant-SOCS7 degrader failed to degrade its target. In contrast, degraders that bound the ALFA tag with nanomolar affinity displayed comparable degradation activity across all cell lines, with the exception of pancreatic cancer cells expressing the chromatin-anchored POI H2B-GFP-ALFA-KRAS^G12V^_166_. In this specific case, the degradation was linked to the sdAbs’ affinity. We excluded the possibility that differences in expression levels of either the biodegraders or the components of the SOCS7-based E3 ligase complex in pancreatic cells could account for these results. In addition, we observed that the POI degradation in pancreatic cancer cells was no longer associated with the affinity of the sdAb when we modified its localization. Hence, this result suggests that the subcellular environment (i.e., nucleus versus cytoplasm) may affect the binding properties of the sdAbs ALFA mutants specifically in pancreatic cancer cells and thus the degradation efficiency of their cognate degraders. Further investigation will be required to decipher the effect of the POI localization on the activity of degraders made of sdAb ALFA mutants in pancreatic cancer cells. In the future, it would be interesting to systematically evaluate whether the same results are obtained with different sdAbs or protein binders and their mutants.

A potential application of our findings would be the use of SOCS7 as an E3 ligase recruited by PROTACs. SOCS7 contains an SH2 domain that serves as a substrate recognition domain mediating binding to phosphorylated partner proteins and a SOCS box that recruits Elongin B/C.^43^ There is currently no available small molecule ligand for SOCS7. Nevertheless, a covalent small molecule ligand targeting a conserved cysteine in SOCS2 has been recently described,^44^ suggesting the feasibility to identify a ligand for SOCS7. Hijacking SOCS7 with PROTACs is an appealing strategy as SOCS7 displays distinct characteristics compared to the widely used E3 ligases, namely VHL and CRBN. While VHL and CRBN are substrate receptors of CRL2 and CRL4, respectively, SOCS7 is a substrate receptor of CRL5. Given that resistance mechanisms often involve the E3 ligase components,^24^ thus the identification of additional E3 ligases from other families, such as SOCS7, could overcome these resistances. Furthermore, SOCS7 has a more tissue-restricted expression compared to the commonly used VHL and CRBN E3 ligases as indicated by the Human Protein Atlas database (https://www.proteinatlas.org/). These differences open up possibilities for a distinct and tailored use of SOCS7-based PROTACs.

Collectively, this study reveals the potential of SOCS7 as novel E3 ligase for TPD. Our findings suggest that SOCS7 should be considered (i) for studies evaluating the functional consequences of a POI’s degradation to provide insights into cellular processes, (ii) for the development of novel PROTACs, provided the discovery of small molecules binding to SOCS7, and also (iii) directly as a component of therapeutic drugs, as exemplified with our SOCS7-based KRAS degrader. As biodegraders do not cross the cell membrane, efficient delivery methods will need to be implemented before considering their application in clinical settings.

### Limitations of the study

In this study, we performed a screening of E3 ligases amenable to TPD, when fused to an anti-ALFA tag sdAb. While we identified three positive hits, we focused our study on SOCS7, the most potent E3 ligase-based biodegrader from the screening. However, this does not imply that RNF144B and FBX38 are not active E3 ligases. Further work could be carried out to better characterize these two E3 ligases, similar to our in-depth analysis of SOCS7. The impact of the linker length was assessed on two SOCS7-based biodegraders and our data demonstrated limited effects on the activity of these biodegraders. Nevertheless, the influence of the linker length may be dependent on many parameters such as the E3 ligase domain and its partners in the E3 complex, the sdAb, the binding sites at the interface between the sdAb and the target or the relative positioning between the E3 ligase domain and the sdAb. Hence, the effect of the linker length should be systematically explored for each novel biodegrader.

## STAR★Methods

### Key resources table

**Table:** 

REAGENT or RESOURCE	SOURCE	IDENTIFIER
**Antibodies**		

FLAG mouse antibody	Sigma	Cat#F1804, RRID:AB_262044
GFP goat antibody	Novus Biologicals	Cat#NB100-1770, RRID:AB_10128178
pan-actin mouse antibody	Sigma	Cat#MAB1501, RRID:AB_2223041
α-tubulin rabbit antibody	Abcam	Cat#ab4074, RRID:AB_2288001
HSP90 rabbit antibody	Cell Signaling Technology	Cat#4874S, RRID:AB_2121214
KRAS mouse antibody	Santa Cruz Biotechnologies	Cat#sc-30, RRID:AB_627865
NRAS goat antibody	Abcam	Cat#ab77392, RRID:AB_1524048
HRAS rabbit antibody	Proteintech	Cat#18295-1-AP, RRID:AB_2121046
Elongin-C rabbit antibody	Abcam	Cat#ab226831
Elongin-B rabbit antibody	Abcam	Cat#ab151743
Cullin 5 rabbit antibody	Abcam	Cat#ab184177
RBX2 rabbit antibody	Abcam	Cat#ab181986
phospho-p44/22 MAPK (pERK1/2) rabbit antibody	Cell Signaling Technology	Cat#4370S, RRID:AB_2315112
p44/42 MAPK (total ERK1/2) rabbit antibody	Cell Signaling Technology	Cat#4695S, RRID:AB_390779
Phospho-AKT S473 rabbit antibody	Cell Signaling Technology	Cat#4058S, RRID:AB_331168
AKT rabbit antibody	Cell Signaling Technology	Cat#9272S, RRID:AB_329827
sdAb anti-ALFA HRP-conjugated antibody	NanoTag Biotechnologies	Cat#N1505v, RRID:AB_3075989
Horse anti-mouse IgG HRP-conjugated antibody	Cell Signaling Technology	Cat#7076, RRID:AB_330924
Goat anti-rabbit IgG HRP-conjugated antibody	Cell Signaling Technology	Cat#7074, RRID:AB_2099233
Chicken anti-goat IgG HRP-conjugated antibody	Immunoreagents	Cat#CkxGt-003-EHRPX
Goat anti-mouse IgG Alexa Fluor 488-conjugated antibody	ThermoFischer Scientific	Cat#A11001, RRID:AB_2534069

**Chemicals, peptides, and recombinant proteins**		

Epoxomicin	Sigma	Cat#E3652
Doxycyline	Sigma	Cat#D9891
Polybrene	Sigma	Cat#107689
Puromycin	Invivogen	Cat#ant-pr-1
Blasticidin	Invivogen	Cat#ant-bl-05
Coelenterazine 400a	Cayman Chemicals	Cat#16157
sdAb ALFA WT-TEV-His	This study	N/A
sdAb ALFA E-TEV-His	This study	N/A
sdAb ALFA SE-TEV-His	This study	N/A
sdAb ALFA SER-TEV-His	This study	N/A
sdAb ALFA SERR-TEV-His	This study	N/A
sdAb ALFA Ctl-TEV-His	This study	N/A
ALFA tag-human Fc	This study	N/A

**Critical commercial assays**		

293fectin Transfection Reagent	ThermoFischer Scientific	Cat#12347-500
JetOptimus	PolyPlus Transfection	Cat#101000006
JetPrime	PolyPlus Transfection	Cat#101000046
Neon™ Transfection System 10 μL Kit MKP1025	ThermoFisher Scientific	Cat#MPK1096
Cell Titer Glo	Promega	Cat#G7572
CellTiter Glo 3D	Promega	Cat#G9683
Protein G magnetic beads	Life Technologies	Cat#10004D
Fluorescence mounting medium	Dako	Cat#S3023
FreeStyle 293 Expression Medium	ThermoFisher Scientific	Cat#12338-018
OptiMEM, no red phenol	ThermoFisher Scientific	Cat# 11058021

**Experimental models: Cell lines**		

HEK293T	ATCC	Cat#CRL-3216, RRID: CVCL_0063
HEK293FS	ThermoFischer Scientific	Cat#R790-07
HeLa S3	ATCC	Cat#CCL-2.2, RRID:CVCL_0058
U2OS	A gift from Dr. O. Sordet	N/A
MIA PaCa-2	ATCC	Cat#CRL-1420, RRID:CVCL_0428
PDAC087T	A gift from Dr. N. Dusetti	N/A
Jurkat	A gift from Prof. B. Segui	N/A

**Recombinant DNA**		

pEF-myc-cyto	Invitrogen	Cat#V89120
TLCV2	Addgene	Cat#87360
TLCV2-FLAG-K19-L5-SOCS7 (L5 = (GGGGS)_1_)	This paper	N/A
TLCV2-FLAG-K19dm-L5-SOCS7	This paper	N/A
pLenti-H2B-GFP-ALFA-KRAS^G12V^_166_	This paper	N/A
pLenti-GFP-ALFA-KRAS^G12V^_166_	This paper	N/A
pEF-3xFLAG-SOCS7	This paper	N/A
pEF-3xFLAG-UBOX	This paper	N/A
pEF-3xFLAG-RNF144B	This paper	N/A
pEF-3xFLAG-VHL	This paper	N/A
pEF-3xFLAG-KLHL7	This paper	N/A
pEF-3xFLAG-sdAbALFA WT	This paper	N/A
pEF-3xFLAG-sdAbGFP4	This paper	N/A
pEF-3xFLAG-sdAbCtl	This paper	N/A
pEF-ALFA-CRAF	This paper	N/A
pEF-RLuc8-L5-ALFA-tag	This paper	N/A
pEF-GFP^2^-L5-sdAbALFA WT	This paper	N/A
pEF-GFP^2^-L5-sdAbALFA E	This paper	N/A
pEF-GFP^2^-L5-sdAbALFA SE	This paper	N/A
pEF-GFP^2^-L5-sdAbALFA SER	This paper	N/A
pEF-GFP^2^-L5-sdAbALFA SERR	This paper	N/A
pEF-GFP^2^-L5-sdAbCtl	This paper	N/A
pEF-FLAG-sdAbALFA WT-L5-SOCS7-IRES-MTS-mCherry	This paper	N/A
pEF-FLAG-sdAbALFA E-L5-SOCS7-IRES-MTS-mCherry	This paper	N/A
pEF-FLAG-sdAbALFA SE-L5-SOCS7-IRES-MTS-mCherry	This paper	N/A
pEF-FLAG-sdAbALFA SER-L5-SOCS7-IRES-MTS-mCherry	This paper	N/A
pEF-FLAG-sdAbALFA SERR-L5-SOCS7-IRES-MTS-mCherry	This paper	N/A
pEF-FLAG-sdAbCtl-L5-SOCS7-IRES-MTS-mCherry	This paper	N/A
pEF-FLAG-sdAbGFP4-L5-SOCS7-IRES-MTS-mCherry	This paper	N/A
pEF-FLAG-K19-L5-SOCS7-IRES-MTS-mCherry	This paper	N/A
pEF-FLAG-K19dm-L5-SOCS7-IRES-MTS-mCherry	This paper	N/A
pEF-FLAG-sdAbALFA WT-L10-SOCS7-IRES-MTS-mCherry (L10 = (GGGGS)_2_)	This paper	N/A
pEF-FLAG-sdAbGFP4-L10-SOCS7-IRES-MTS-mCherry	This paper	N/A
pEF-FLAG-sdAbCtl-L10-SOCS7-IRES-MTS-mCherry	This paper	N/A
pEF-FLAG-sdAbALFA WT-L15-SOCS7-IRES-MTS-mCherry (L15 = (GGGGS)_3_)	This paper	N/A
pEF-FLAG-sdAbGFP4-L15-SOCS7-IRES-MTS-mCherry	This paper	N/A
pEF-FLAG-sdAbCtl-L15-SOCS7-IRES-MTS-mCherry	This paper	N/A
pEF-SOCS7-L5-sdAbGFP4-FLAG-IRES-MTS-mCherry	This paper	N/A
pEF-SOCS7-L5-sdAbALFA WT-FLAG-IRES-MTS-mCherry	This paper	N/A
pEF-SOCS7-L5-sdAbCtl-FLAG-IRES-MTS-mCherry	This paper	N/A
pEF-VHL-L5-sdAbGFP4-FLAG-IRES-MTS-mCherry	This paper	N/A
pEF-VHL-L5-sdAbALFA WT-FLAG-IRES-MTS-mCherry	This paper	N/A
pEF-VHL-L5-sdAbCtl-FLAG-IRES-MTS-mCherry	This paper	N/A
pEF-KLHL7-L5-sdAbGFP4-FLAG-IRES-MTS-mCherry	This paper	N/A
pEF-KLHL7-L5-sdAbALFA WT-FLAG-IRES-MTS-mCherry	This paper	N/A
pEF-KLHL7-L5-sdAbCtl-FLAG-IRES-MTS-mCherry	This paper	N/A
pEF-FLAG-sdAbGFP4-L5-SPOP-IRES-MTS-mCherry	This paper	N/A
pEF-FLAG-sdAbALFA WT-L5-SPOP-IRES-MTS-mCherry	This paper	N/A
pEF-FLAG-sdAbCtl-L5-SPOP-IRES-MTS-mCherry	This paper	N/A
pEF-FLAG-sdAbGFP4-L5-UBOX-IRES-MTS-mCherry	This paper	N/A
pEF-FLAG-sdAbALFA WT-L5-UBOX-IRES-MTS-mCherry	This paper	N/A
pEF-FLAG-sdAbCtl-L5-UBOX-IRES-MTS-mCherry	This paper	N/A
pEF-RNF144B-L5-sdAbALFA WT-FLAG-IRES-MTS-mCherry	This paper	N/A
pEF-FBX38-L5-sdAbALFA WT-FLAG-IRES-MTS-mCherry	This paper	N/A
pEF-FBXW8-L5-sdAbALFA WT-FLAG-IRES-MTS-mCherry	This paper	N/A
pEF-CDC20-L5-sdAbALFA WT-FLAG-IRES-MTS-mCherry	This paper	N/A
pEF-FLAG-sdAbALFA WT-L5-RNF144B-IRES-MTS-mCherry	This paper	N/A
pEF-FLAG-sdAbALFA WT-L5-FEM1B-IRES-MTS-mCherry	This paper	N/A

**Software and algorithms**		

GraphPad Prism 9.0	GraphPad Prism software	N/A
FlowJo 10	FlowJo software	N/A
Fiji	ImageJ	N/A

**Other**		

White 96 well plate	Greiner	Cat#655075
Ultra-low attachment 96-well plates	Corning	Cat#7007
White 96 well plate, clear bottom	Revvity	Cat#6005181
Sartolab RF 50 PES sterile vacuum filtration units	Sartorius	Cat#180E01

### Resource availability

#### Lead contact

Further information and requests for resources and reagents should be directed to and will be fulfilled by the Lead Contact, Nicolas Bery at Nicolas.bery@inserm.fr.

#### Materials availability

Plasmids generated in this study, which are in detail described in the “STAR methods”. The plasmids are available from the lead contact with a completed Materails Transfer Agreement.

#### Data and code availability

• Data: All the data reported in this study will be shared by the lead contact upon request.
• Code: This paper does not report original code.
• Additional information: Any additional information required to reanalyse the data reported in this paper is available from the lead contact upon request.

### Experimental model and study participant details

#### Cell culture

HEK293T, HeLa S3, U2OS, MIA PaCa-2, PDAC087T cells were grown in DMEM medium (Life Technologies) and Jurkat cells in RPMI medium (Life Technologies). All cell lines were supplemented with 10% FBS (Sigma) and 1% Penicillin/Streptomycin (Life Technologies). Cells were grown at 37°C with 5% CO_2_. All cell lines were tested to confirm that they were free of mycoplasma.

### Method details

#### Cell transfection

Cells were seeded in 6 well plates for Western blotting experiments or 12 well plates for flow cytometry (170,000 cells for PDAC087T; 120,000 cells for HEK293T cells; 80,000 cells for MIA PaCa-2 and 60,000 for all the other cell lines) and were transfected 24 h later with JetOptimus (PolyPlus Transfection, Cat#101000006) for PDAC087T or JetPrime (PolyPlus Transfection, Cat#101000046, see also BRET2 section) for all the other cell lines: 2 μg of plasmid +4 μL of JetPrime in 200 μL of JetPrime buffer (Western blot) or 0.8 μg of plasmid +1.6 μL of JetPrime in 80 μL of JetPrime buffer (flow cytometry). The medium was changed 4 h after transfection. Cells were incubated for another 48 h before flow cytometry or Western blotting analyses.

Jurkat cells (2 × 10^7^ cells/mL, in 25 μL of R electroporation buffer) were electroporated with the Neon system (ThermoFisher Scientific, Cat#MPK1096) with the following settings: 3.25 μg of plasmid DNA, 1325 V, 10 ms, 3 pulses, 10 μL tips and then added to 2 mL of medium without antibiotics in a 6 well plate. Cells were incubated for another 48 h before flow cytometry analysis.

#### Cell treatment

Epoxomicin (Sigma, Cat#E3652) was used at 0.8 μM for 20 h in the proteasome inhibition experiments. Doxycyline (Sigma, Cat#D9891) was used to induce the expression of FLAG-K19-SOCS7 or FLAG-K19dm-SOCS7 from the TLCV2 lentivector.

#### Molecular cloning

##### Degrader vectors construction

pEF-VHL-L5-sdAbGFP4-FLAG, pEF-FLAG-sdAbGFP4-L5-SPOP and pEF-FLAG-sdAbGFP4-L5-UBOX plasmids generation: VHL (full-length), L5-SPOP (amino acids 167–374) and L5-UBOX (amino acids 128–303 from the CHIP E3 ligase) were amplified by PCR and inserted into pEF-myc-cyto plasmid (Invitrogen) with PmlI/XhoI sites (VHL) or NotI/XbaI sites (SPOP and UBOX). GGGGS/L5 linker was added between XhoI and NotI sites for VHL-based plasmid. A single FLAG tag was added by PCR at the carboxy-terminal end (NotI/XbaI sites) or on the amino terminal end (PmlI/NcoI sites) of the sdAb degrader vectors. sdAb GFP4^37^ was inserted with NcoI/NotI sites into pEF-VHL-L5-MCS-FLAG, pEF-FLAG-MCS-L5-SPOP and pEF-FLAG-MCS-L5-UBOX plasmids.

Gene blocks for the E3 ligases (RNF144B, FBX38, FBXW8, KLHL7, CDC20, SOCS7 and FEM1B) were obtained from IDT. N-terminal E3 ligases (RNF144B, FBX38, FBXW8, KLHL7 and CDC20) were cloned between PmlI/XhoI sites of the pEF-VHL-L5-sdAbGFP4-FLAG plasmid, replacing the VHL moiety. For C-terminal E3 ligases (RNF144B, SOCS7 and FEM1B), they were cloned between NotI/XbaI sites of the pEF-FLAG-sdAbGFP4-L5-SPOP plasmid replacing the SPOP moiety. Similarly, N-terminal SOCS7 was cloned by PCR between PmlI/XhoI sites of the pEF-VHL-L5-sdAbGFP4-FLAG plasmid. (GGGGS)_2_ and (GGGGS)_3_ linkers were cloned between NotI/SalI sites and SOCS7 was amplified by PCR and inserted between SalI/XbaI sites of the pEF-FLAG-NbALFA-L5-SOCS7 plasmid digested by NotI/XbaI to generate pEF-FLAG-NbALFA-(GGGGS)_2_-SOCS7 and pEF-FLAG-NbALFA-(GGGGS)_3_-SOCS7 plasmids.

The sequence IRES-MTS-mCherry^5^ was added with single digest XbaI on the carboxy-terminal end of each degrader vector.

sdALFA WT (gene synthesis, IDT) and sdAb Ctl were cloned between NcoI/NotI of the amino and carboxy-terminal E3 ligases-IRES-MTS-mCherry vectors.

DARPins K19 and K19dm (gene synthesis, IDT) were cloned into the pEF-FLAG-sdAbALFA-L5-SOCS7-IRES-MTS-mCherry vector between NcoI/NotI sites replacing the sdAb ALFA moiety.

##### Generation of the sdALFA mutants

The generation of sdAb ALFA mutants was performed by PCR site-directed mutagenesis, using pEF-FLAG-sdAb ALFA-L5-SOCS7 as template. The following mutations were introduced: sdAb ALFA E58S (E), sdAb ALFA S57A/E58S (SE), sdAb ALFA S57A/E58S/R59G (SER), sdAb ALFA S57A/E58S/R59G/R65D (SERR) and sdAb ALFA S57A/E58S/R59G/R65D/D112V (SERRD or Ctl). sdAb ALFA mutants were cloned by PCR between NotI/XbaI of the BRET vector pEF-GFP^2^-MCS^45^ and between NcoI/NotI sites in pEF-FLAG-MCS-L5-SOCS7-IRES-MTS-mCherry plasmid.

##### Lentivectors construction

###### *FLAG-K19-SOCS7* and *FLAG-K19dm-SOCS7*

Sequences were inserted between AgeI/NheI sites of TLCV2 lentivector (Addgene plasmid #87360)^46^ by PCR.

###### H2B-GFP-ALFA-KRAS^G12V^_166_ plasmid construction

pEF-ALFA-KRAS^G12V^_166_ was first generated by inserting ALFA tag (PmlI/NotI sites) and KRAS^G12V^_166_ (NotI/XbaI sites) into pEF-myc-cyto plasmid. pEF-H2B-GFP-ALFA-KRAS^G12V^_166_ was generated by inserting H2B-GFP (gene synthesis, IDT) and ALFA-KRAS^G12V^_166_ into pEF-MCS by PCR using PmlI/SalI and SalI/XbaI sites, respectively. Gateway technology (Invitrogen, Cat#11789 and #11791) was used to obtain the pLenti-H2B-GFP-ALFA-KRAS^G12V^_166_ plasmid from the pEF-H2B-GFP-ALFA-KRAS^G12V^_166_ plasmid.

###### GFP-ALFA-KRAS^G12V^_166_ plasmid construction

The plasmid construct of pEF-H2B-GFP-ALFA-KRAS^G12V^_166_ was digested with NcoI/XbaI to extract the insert GFP-ALFA-KRAS^G12V^_166_. This insert was cloned into the pEF-myc-cyto plasmid. Gateway technology was used to obtain the pLenti-GFP-ALFA-KRAS^G12V^_166_ plasmid from the pEF-GFP-ALFA-KRAS^G12V^_166_ plasmid.

##### 3xFLAG vectors construction

SOCS7, SPOP, UBOX, RNF144B, sdAb ALFA WT, sdAb Ctl and sdAb GFP4 were digested with NotI/XbaI and inserted in the pEF-3xFLAG-MCS.^16^ VHL and KLHL7 were cloned into the pEF-3xFLAG-MCS by PCR using NotI/XbaI sites.

##### ALFA-CRAF vector construction

pEF-ALFA-KRAS^G12V^_166_ was digested with NotI/XbaI and full-length CRAF was then inserted in this digested plasmid using NotI/XbaI sites to generate pEF-ALFA-CRAF.

##### BRET vectors construction

The different sdAbs were cloned between NotI/XbaI sites of the pEF-GFP^2^-MCS and the ALFA tag between XhoI/XbaI sites of the pEF-RLuc8-MCS^45^ to obtain pEF-GFP^2^-L5 (i.e., GGGGS)-sdAbs and pEF-RLuc8-L5-ALFA-tag.

The DNA coding sequences of the biodegraders and the target antigens are available in Table S1.

#### Cell transduction

Cells were transduced with the appropriate lentiviral particles for 36 h in 6 well-plate in 1 mL of medium containing 8 μg mL^−1^ of polybrene (Sigma, Cat#107689). Transduced cells were selected with puromycin (Invivogen, Cat#ant-pr-1) or blasticidin (Invivogen, Cat#ant-bl-05).

#### BRET2 saturation assays

280,000 HEK293T were seeded per well of a 12 well plates and incubated for 24 h at 37°C. Cells were then transfected with a total of 640 ng of DNA mix, containing the donor + acceptor plasmids, using JetPrime transfection reagent (PolyPlus Transfection). After 24 h, cells were detached, washed with PBS and seeded in a white 96 well plate (clear bottom, Revvity, Cat#6005181) in OptiMEM no phenol red medium (ThermoFisher Scientific, Cat#11058021) complemented with 4% FBS. Cells were incubated for an additional 24 h at 37°C before the BRET assay reading. A detailed protocol is provided elsewhere.^45^

#### BRET2 measurements

Total GFP^2^ fluorescence was first detected with excitation and emission peaks set at 400 nm ± 15 and 510 nm ± 20 respectively. Then, the BRET signal was detected as follow: coelenterazine 400a substrate (10 μM final) was injected into cells (Cayman Chemicals, Cat#16157) with a CLARIOstar instrument (BMG Labtech) using a luminescence module. Finally, total RLuc8 luminescence was measured with an emission peak set at 480 nm ± 80 by reinjecting coelenterazine 400a substrate at 10 μM final concentration.

The BRET ratio corresponds to the light emitted by the GFP^2^ acceptor constructs (515 nm ± 30) upon addition of coelenterazine 400a divided by the light emitted by the RLuc8 donor constructs (410 nm ± 80). The background signal is subtracted from that BRET ratio using the donor-only negative control where only the RLuc8 plasmid is transfected into the cells. Total GFP^2^ and RLuc8 signals were used to control the protein expression from each plasmid.

#### Flow cytometry

To quantify GFP fluorescence intensity, cells were washed with 1xPBS, detached with trypsin and resuspended in 1xPBS-4% FBS and analyzed on a MACSQuant VYB flow cytometer (Miltenyi). The instrument settings log forward scatter (FSC) and log side scatter (SSC) were adapted to the size of the cells. The singlet cell population was gated on FSC and SSC. The typical sample size was 20,000 singlets or 3,000 mCherry positive cells per measurement depending on the cell line. The data were analyzed with FlowJo software. The median fluorescence intensity (MFI) was analyzed in the mCherry transfected cells and the results are given with a normalized GFP MFI (MFI of the degrader/MFI of the negative control x 100).

#### Immunofluorescence

Cells plated on a coverslip were transfected the next day with plasmids expressing 3xFLAG-E3 ligases for 24 h. To visualize the expression of the E3 ligases, cells were washed with PBS, fixed with 4% paraformaldehyde (PFA) in 1xPBS, permeabilized with 0.2% Triton in 1xPBS and blocked in 8% Bovine Serum Albumin (BSA) in 1xPBS for 1 h. Then, cells were incubated with primary anti-FLAG antibody (1/2000, Sigma, Cat#F1804) in 5% BSA-1xPBS for 1 h at room temperature (RT, 25°C). After 3 washes with 1xPBS, secondary anti-mouse antibody coupled to Alexa Fluor 488 diluted in 5% BSA-1xPBS (1/1000, ThermoFischer Scientific, Cat#A11001) was added for 2 h at RT. Nucleus were marked with 4′,6-diamidino-2-phenylindole (DAPI) and coverslips were mounted using a fluorescence mounting medium (Dako, Cat#S3023). Images were obtained using a confocal LSM880 Airyscan microscope (Zeiss) and analyzed with Fiji software.

#### VHH and antigen expression and purification

The plasmids encoding the VHHs ALFA and mutants and the ALFA-tag-human-Fc were transiently transfected into HEK293FS cells (ThermoFischer Scientific, Cat#R790-07) with 293fectin Transfection Reagent (ThermoFischer Scientific, Cat#12347-500) in OptiMEM medium (ThermoFischer Scientific, Cat#11058-021). After 30 min of incubation, the cells were transferred to 50 mL spintubes (ThermoFischer Scientific, Cat#12590457) in FreeStyle 293 Expression Medium (ThermoFisher Scientific, Cat#12338-018) and grown at 37°C with 8% CO_2_. After 7 days, the cells were harvested by centrifuging the spintubes. The supernatants were filtered on Sartolab RF 50 PES sterile vacuum filtration units (Sartorius, Cat#180E01). The VHHs in the supernatants were purified thanks to their 6xHis tag by Immobilized Metal Chelate Affinity Chromatography (IMAC) on column on a Protein Maker (Protein BioSolutions). Elution was realized with 250 mM of imidazole. Buffer exchange was then realized with 1xDPBS and a sterile filtration was performed. For the antigen ALFA-tag-human-Fc protein, a Protein G affinity chromatography was performed, as well as buffer exchange and sterile filtration.

#### Affinities determination

Surface Plasmon Resonance (SPR) experiments were performed on a Biacore T200 biosensor instrument at RT on a sensor chip CM5 with anti-human Fc covalently immobilized by amine coupling. The ligand ALFA-Fc was diluted to a concentration of 0.25 μg mL^−1^ in running buffer HBS-EP+ and injected over the chip at a flow rate of 10 μL min^−1^ for 10 min. The analytes (sdAb ALFA and mutants) were diluted in series in running buffer and injected over the chip surface at a flow rate of 30 μL min^−1^. Association was followed for 5 min and dissociation was followed for up to 30 min. The surface was regenerated with one pulse of 30 s of 3 mol.L^−1^ MgCl_2_ (10 μL min^−1^). All sensorgrams are doubled referenced with reference flowcell and blank injection of buffer.

#### NanoDSF

The prometheus NT.48 instrument (NanoTemper technologies) was used to determine the melting temperatures. The samples were loaded into nanoDSF grade Standard Capillaries and analyzed using PrThermoControl, the Prometheus NT.48 nanoDSF software (NanoTemper technologies). The capillaries were filled with 10 μL sample and placed on the sample holder. A temperature gradient of 1 °C min^−1^ from 20°C to 90°C was applied with an excitation power of 100%. Samples are duplicated. The intrinsic protein fluorescence at 330 and 350 nm was recorded. The results were processed on the PR Stability Analysis software (NanoTemper technologies).

#### 2D and 3D cell proliferation assays

1,000 MIA PaCa-2 cells were seeded in white 96 well plates (clear bottom, Revvity, Cat#6005181) for 2D proliferation assays or in ultra-low attachment 96 well plates (Corning, Cat#7007) for 3D spheroid assays. The cell seeding was optimized to maintain linear growth over the time of the assay. The following day doxycycline was prepared at 10X concentration (5 μg mL^−1^ for 0.5 μg mL^−1^ final concentration). Cells were incubated in the presence of the doxycycline for 6 days. Cell viability was determined every two days using CellTiter-Glo (Promega, Cat#G7572) or 3D CellTiter-Glo (Promega, Cat#G9683) by incubation with the cells for 15 min. Cell viability was determined by normalizing doxycycline treated cells to non-treated cells at day 6. Cells from the ultra-low attachment plates were transferred into a white 96 well plate (Greiner, Cat#655075). Plates were read on a CLARIOstar instrument.

#### Immuno-precipitation assay

HEK293T cells were transfected with plasmids that expressed 3xFLAG-tagged sdAbs and ALFA-CRAF for 24 h. Cells were washed with PBS and lyzed with the immuno-precipitation buffer (150 mM NaCl, 50 mM Tris-HCl pH 7.4, 10 mM MgCl_2_, 10% glycerol and 0.5% Triton X-100) supplemented with protease inhibitors (Sigma, Cat#P8340) and phosphatase inhibitors (Thermo-Fisher, Cat#1862495) for 20 min. Immunoprecipitation was performed with anti-FLAG antibody (Sigma, Cat#F1804) and protein G magnetic beads (Life Technologies, Cat#10004D) 4 h at 4°C on a wheel. Beads were washed 5 times with the IP buffer, the bound proteins were eluted with 1X loading buffer and resolved on 12.5% sodium dodecyl sulfate polyacrylamide gel electrophoresis (SDS-PAGE).

#### Western Blot analysis

Cells were washed once with PBS and lyzed in radioimmunoprecipitation assay buffer (RIPA) (50 mM Tris-HCl pH 7.4, 150 mM NaCl, 1% Nonidet 40 (NP40), 1% NaDoc and 1% SDS) or SDS-Tris buffer (1% SDS, 10 mM Tris-HCl pH 7.4) supplemented with protease inhibitors (Sigma) and phosphatase inhibitors (Thermo-Fisher). Cell lysates were sonicated with a Branson Sonifier and the protein concentrations determined by using either BC assay (Interchim, Cat#UP40840A) or the Bio-Rad Protein Assay Dye Reagent (Bio-Rad, Cat#5000006). Equal amounts of protein (20–60 μg) were resolved on 10–15% SDS-PAGE and subsequently transferred onto nitrocellulose membranes using the Trans-Blot Turbo semidry system (Bio-Rad). The membrane was blocked with 10% non-fat milk (Sigma, Cat#70166) or 10% BSA (Euromedex, Cat#04100812E) in TBS-0.1% Tween 20 and incubated overnight with primary antibody at 4°C. After washing the membrane was incubated with HRP conjugated secondary antibody for 1 h at RT. The membrane was washed with TBS-0.1% Tween 20 and developed using Clarity Western ECL Substrate (Bio-Rad) and the ChemiDoc XRS+ imaging system (Bio-Rad).

Primary antibodies include anti-GFP (1/1000, Novus, Cat#NB100-1770), anti-FLAG (1/2000, Sigma, Cat#F1804), anti-actin (1/50000, Sigma, Cat#MAB1501), anti-α-tubulin (1/2000, Abcam, Cat#ab4074), anti-HSP90 (1/4000, CST, Cat#4874S), anti-KRAS (1/100, Santa Cruz Biotechnologies, Cat#sc-30), anti-NRAS (1/1000, Abcam, Cat#ab77392), anti-HRAS (1/500, Proteintech, Cat#18295-1-AP), anti-ALFA HRP-linked (1/4000, NanoTag Biotechnologies, Cat#N1505), anti-Elongin-C (1/1000, Abcam, Cat#ab226831), anti-Elongin-B (1/1000, Abcam, Cat#ab151743), anti-Cullin 5 (1/1000, Abcam, Cat#ab184177), anti-RBX2 (1/1000, Abcam, Cat#ab181986), anti-phospho-p44/22 MAPK (ERK1/2) (1/1000, CST, Cat#4370S), anti-p44/42 MAPK (total ERK1/2) (1/1000, CST, Cat#4695S), anti-phospho-AKT S473 (1/1000, CST, Cat#4058S) and anti-AKT (1/1000, CST, Cat#9272S). Secondary antibodies include anti-mouse IgG HRP-linked (CST, Cat#7076), anti-rabbit IgG HRP-linked (CST, Cat#7074), anti-goat IgG HRP-linked (Immunoreagents, Cat#CkxGt-003-EHRPX).

### Quantification and statistical analysis

BRET titration curves and statistical analysis were performed using Prism 9.0 (GraphPad Software). Values reported represent mean ± standard deviation (SD) of at least three independent experiments. Statistical analyses were performed with a one-way ANOVA or an unpaired t-test as specified in the figure legends. ∗*p* < 0.05, ∗∗*p* < 0.01, ∗∗∗*p* < 0.001, ∗∗∗∗*p* < 0.0001, ns: not significant.

## Supplemental information

Document S1. Figures S1–S9

Table S1. DNA coding sequences of all constructs, related to STAR Methods
