# Supplementary material for: Discovery of SOCS7 as a versatile E3 ligase for protein-based degraders
Source: iScience. 2024 Apr 23;27(5):109802. doi: 10.1016/j.isci.2024.109802 (PMC11090907; doi:10.1016/j.isci.2024.109802)
Supplement: Document S1. Figures S1–S9 [file mmc1.pdf]

iScience, Volume *27*

Supplemental information

Discovery of SOCS7 as a versatile E3 ligase for protein-based degraders

Anaïs Cornebois, Marie Sorbara, Margot Cristol, Emmanuelle Vigne, Pierre Cordelier, Klervi Desrumeaux, and Nicolas Bery

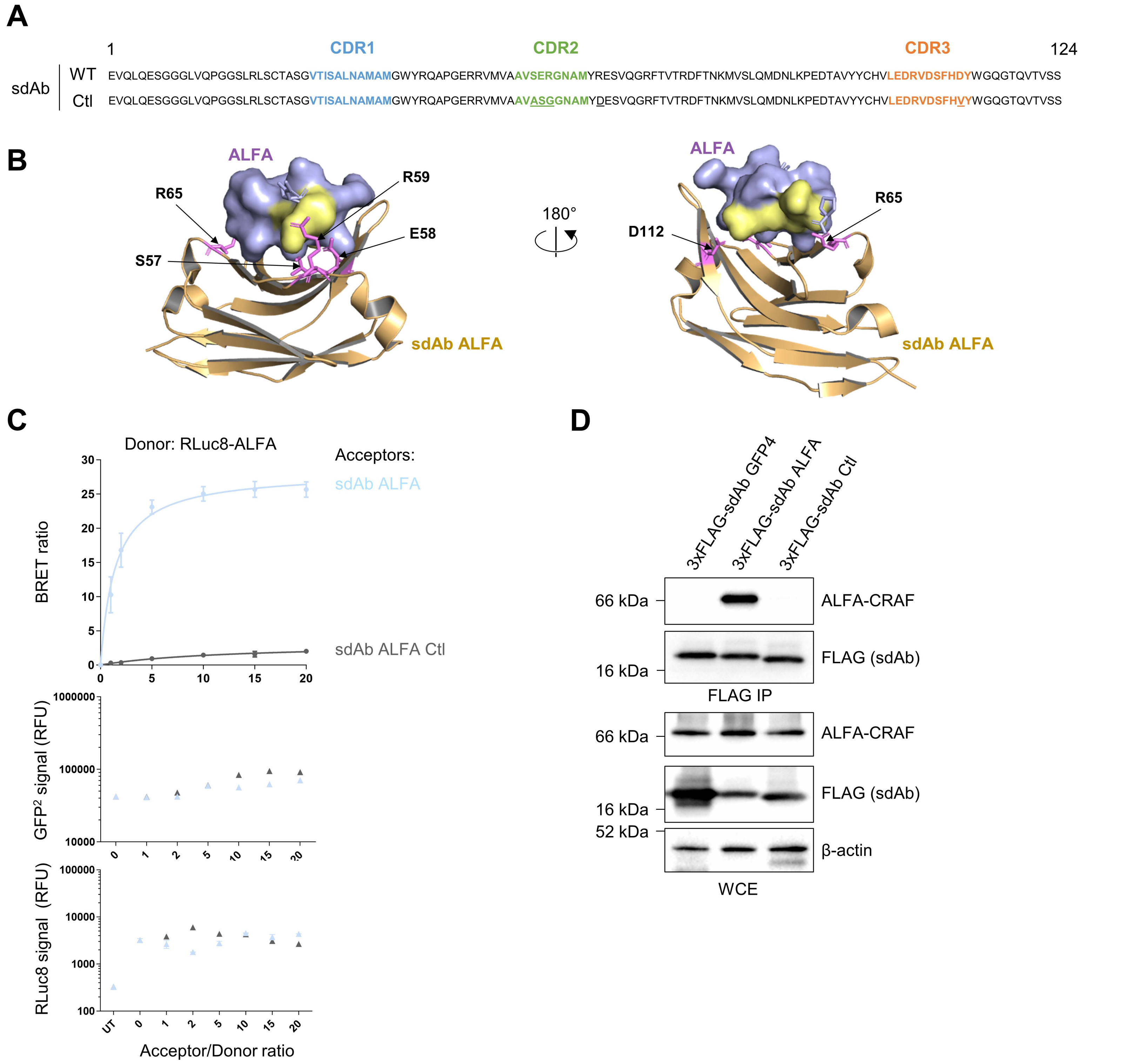

Figure S1: Development of a negative control sdAb derived from anti-ALFA sdAb, related to Figure 1. (A) Amino acid sequence alignment of the sdAb ALFA WT and the control sdAb (Ctl, non-binding mutant). The framework is in black, the complementarity-determining regions (CDRs) are coloured in blue (CDR1), green (CDR2) and orange (CDR3). Amino acids mutated in sdAb Ctl are underlined. (B) Localisation of sdAb Ctl mutations (S57A, E58S, R59G, R65D and D112V) are displayed in magenta on the parental sdAb ALFA structure (shown in wheat coulour) with the affected ALFA residues shown in yellow on ALFA structure (displayed in purple). sdAb ALFA-ALFA tag structure used is PDB 6I2G. (C) BRET donor saturation assay between RLuc8-ALFA (donor) and the acceptors sdAb ALFA WT (light blue) and sdAb ALFA Ctl (grey) with total GFP^2 and RLuc8 signal controls. (D) Co-immunoprecipitation of ALFA-CRAF with the 3xFLAG-sdAb fusions in HEK293T cells. IP: Immuno-Precipitation, WCE: Whole Cell Extract. Error bars in (C) correspond to mean values ± standard deviation (SD) of two independent biological repeats. A representative experiment out of two independent biological repeats is shown in panel D.

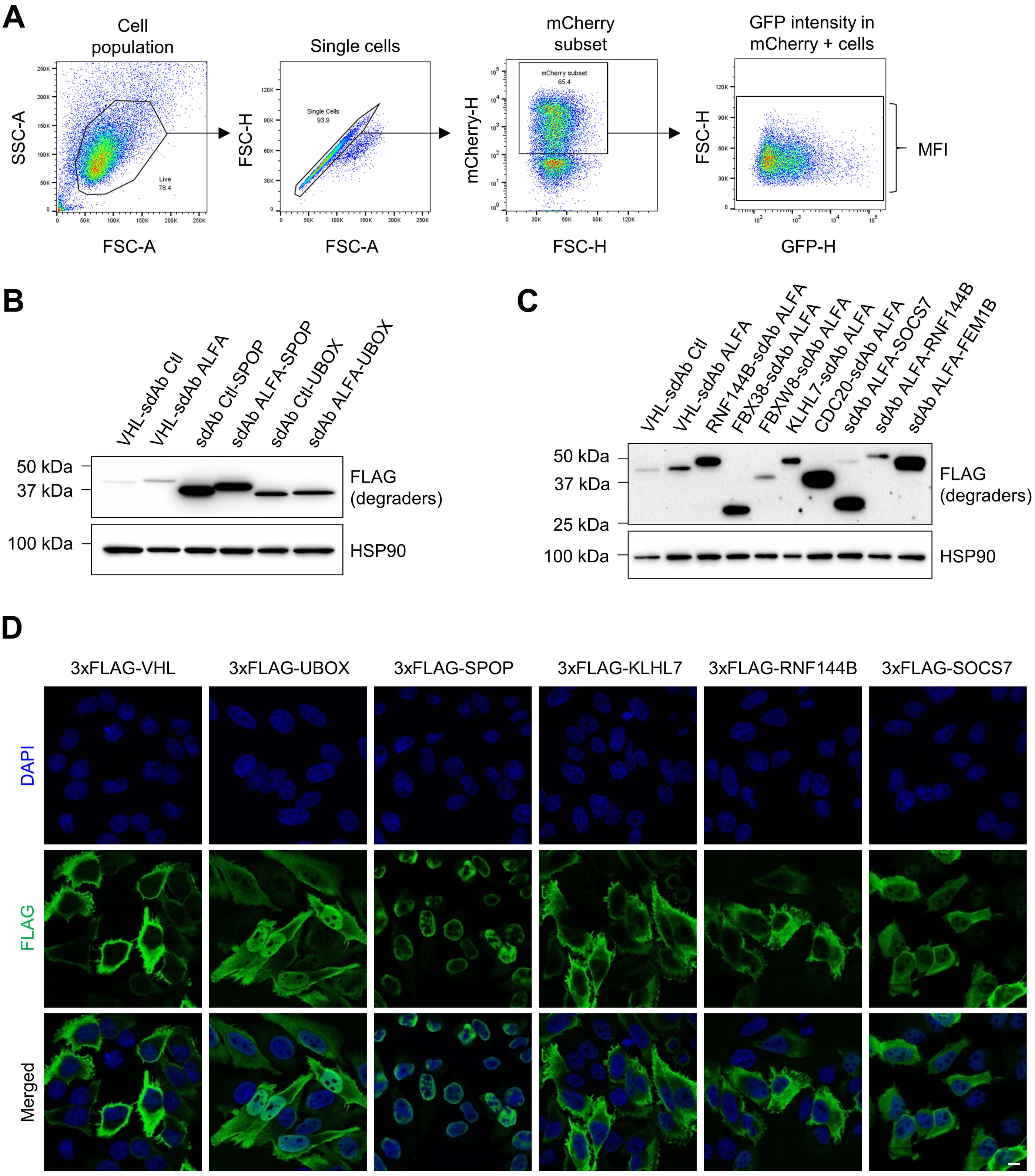

Figure S2: Characterisation of the different E3 ligases screened, related to Figure 1. (A) Gating strategy for the acquisition of flow cytometry data. FSC: forward scatter, SSC: side scatter, MFI: median fluorescence intensity. (B, C) Expression level of each biodegrader used in Figure 1B (B) or in Figure 1C (C) is assessed by Western blot and detected by a FLAG antibody. HSP90 is the loading control. (D) Confocal images of HeLa S3 transiently transfected with indicated 3xFLAG-E3 ligase constructs detected with a FLAG antibody. DAPI is used as a nucleus dye. Scale bar: 10 μm. Each experiment was performed twice (B, C) or three times (D) as independent biological repeats.

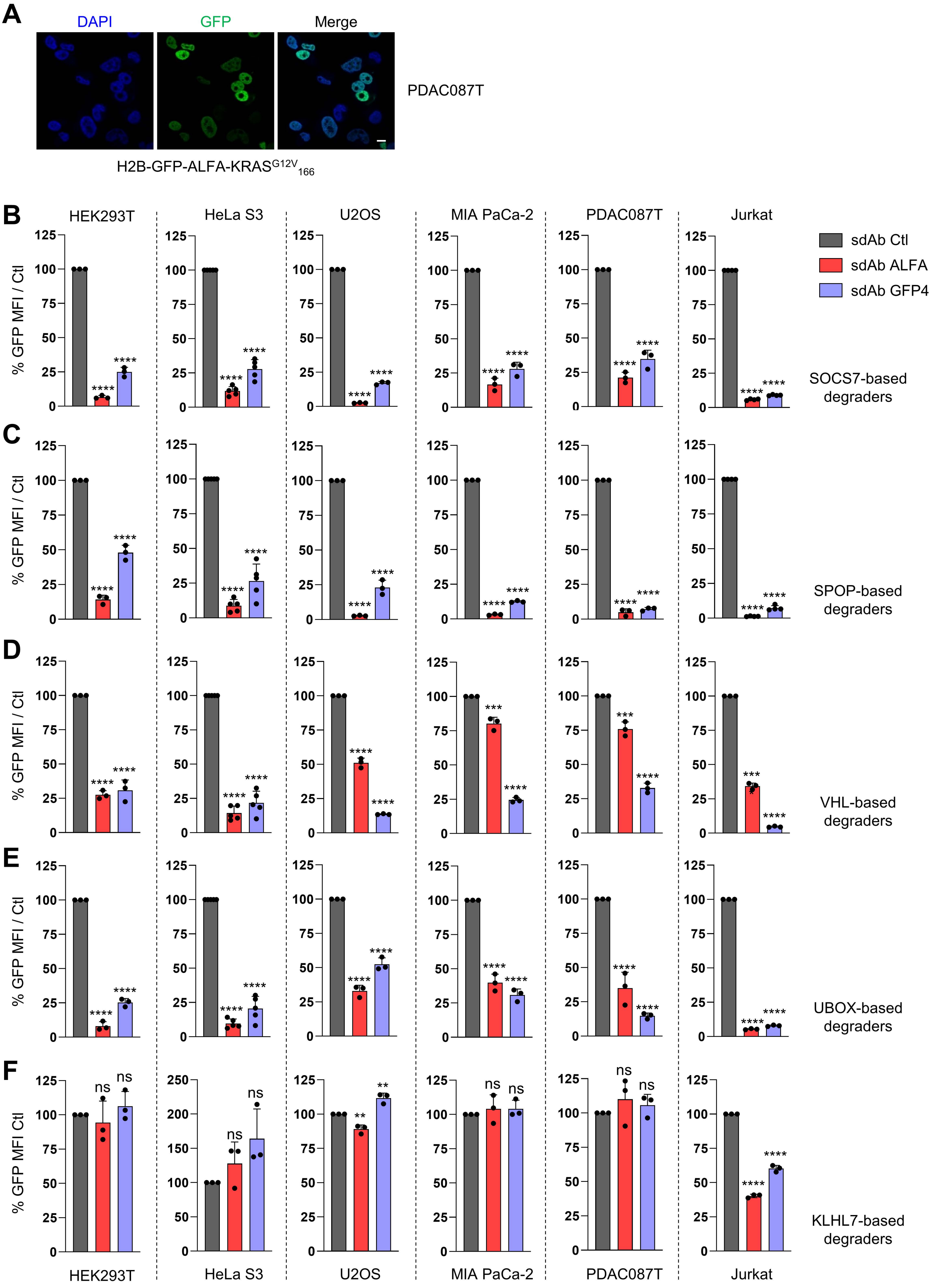
A
DAPI
GFP
Merge
PDAC087T
H2B-GFP-ALFA-KRAS^{G12V}$_{166}$
B
C
D
E
F
HEK293T
HeLa S3
U2OS
MIA PaCa-2
PDAC087T
Jurkat
% GFP MFI / Ctl
% GFP MFI Ctl
sdAb Ctl
sdAb ALFA
sdAb GFP4
SOCS7-based degraders
SPOP-based degraders
VHL-based degraders
UBOX-based degraders
KLHL7-based degraders
****
***
**
ns

Figure S3: Degradation efficacy of E3 ligase-based biodegraders in different cell lines expressing H2B-GFP-ALFA-KRAS$^{G12V}_{166}$, related to Figure 2. (A) Representative field of fixed PDAC087T cells stably expressing H2B-GFP-ALFA-KRAS$^{G12V}_{166}$. Scale bar: 10 μm. (B-F) Normalised GFP MFI data from Figure 2. Six different cell lines expressing H2B-GFP-ALFA-KRAS$^{G12V}_{166}$ are transfected with sdAb ALFA (red bars), GFP4 (purple bars) or control (grey bars)-based degraders. (B) SOCS7, (C) SPOP, (D) VHL, (E) UBOX and (F) KLHL7-based biodegraders' data. Statistical significance was determined by one-way ANOVA followed by Dunnett post-hoc tests in (B-F) (**p<0.01; ***p<0.001; ****p<0.0001; ns, not significant). Error bars in (B-F) are mean ± SD of at least three independent biological repeats.

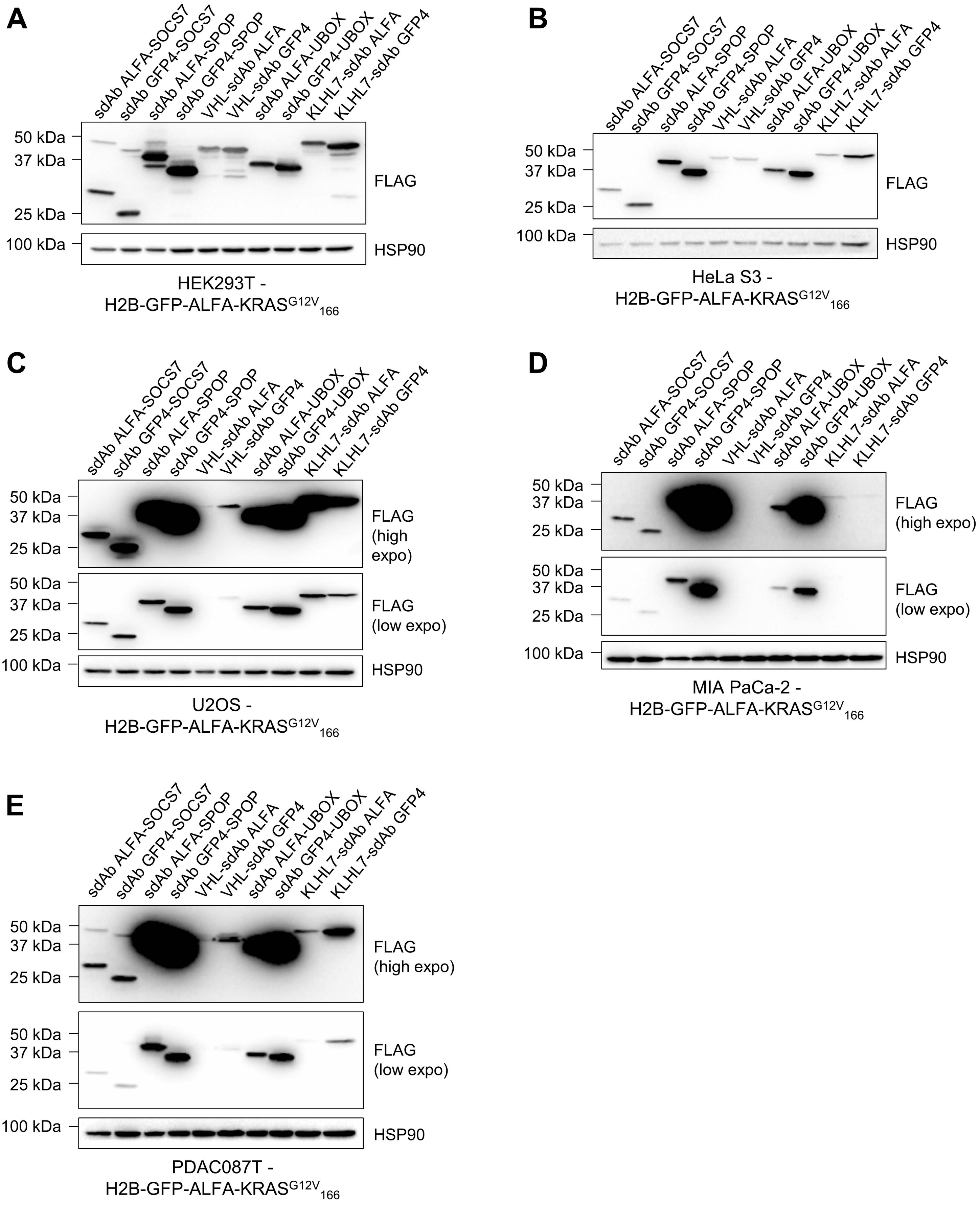

Figure S4: Control of expression of E3 ligase-based biodegraders in cell lines expressing H2B-GFP-ALFA-KRAS$^{G12V}{}_{166}$, related to Figure 2. Western blot analyses to control the expression level of the biodegraders (FLAG tag) from Figure 2 in HEK293T (A), HeLa S3 (B), U2OS (C), MIA PaCa-2 (D) and PDAC087T (E) cell lines stably expressing H2B-GFP-ALFA-KRAS$^{G12V}{}_{166}$. HSP90 is the loading control. High/low expo: high/low exposure. A representative experiment out of two independent biological repeats is shown in panel (A-E).

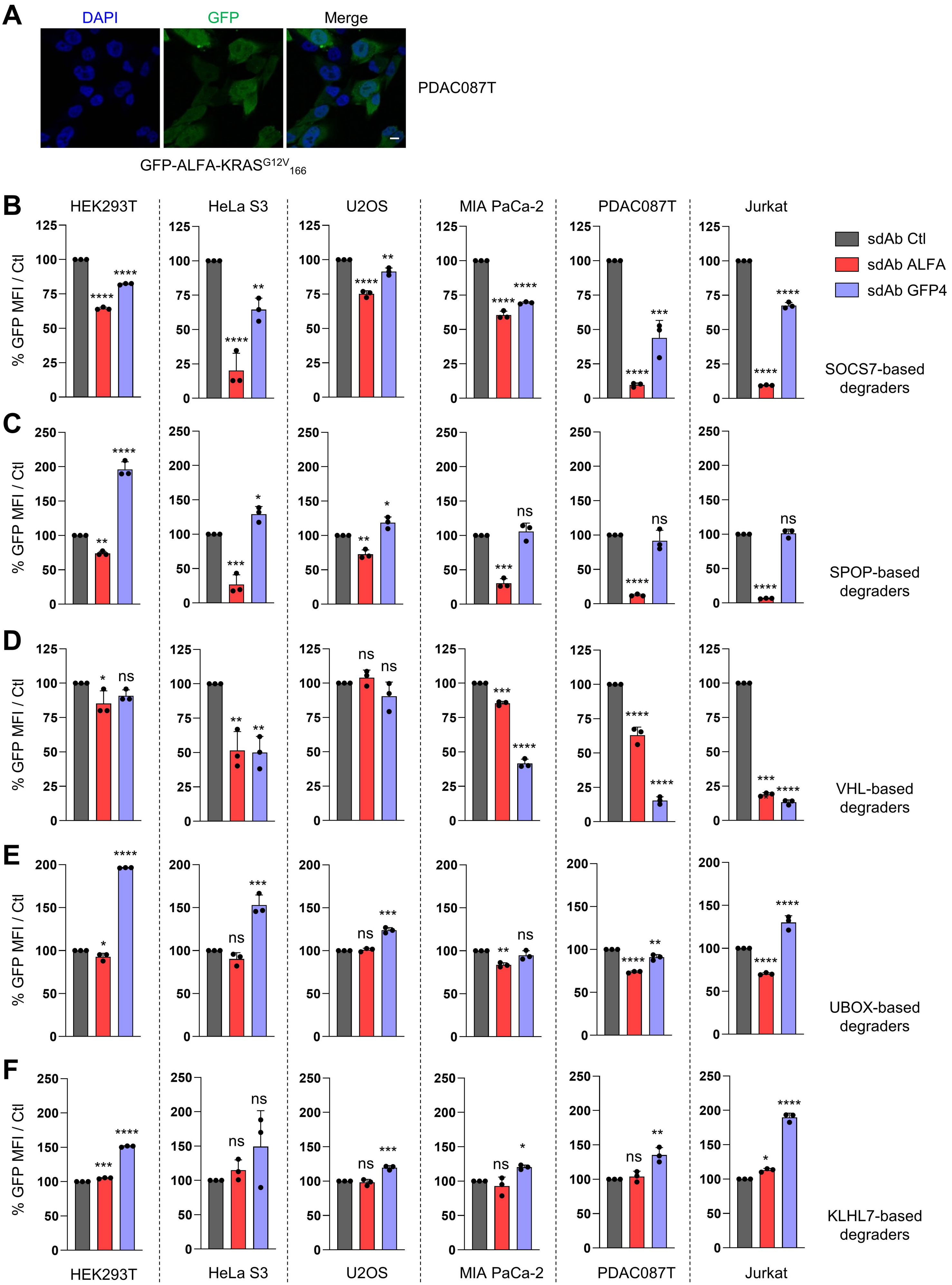
A
DAPI
GFP
Merge
PDAC087T
GFP-ALFA-KRASG12V166
B
C
D
E
F
HEK293T
HeLa S3
U2OS
MIA PaCa-2
PDAC087T
Jurkat
% GFP MFI / Ctl
sdAb Ctl
sdAb ALFA
sdAb GFP4
SOCS7-based degraders
SPOP-based degraders
VHL-based degraders
UBOX-based degraders
KLHL7-based degraders

Figure S5: Degradation efficacy of E3 ligase-based biodegraders in different cell lines expressing GFP-ALFA-KRAS$^{G12V}_{166}$, related to Figure 3. (A) Representative field of fixed PDAC087T cells stably expressing GFP-ALFA-KRAS$^{G12V}_{166}$. Scale bar: 10 μm. (B-F) Normalised GFP MFI data from Figure 3. Six different cell lines expressing GFP-ALFA-KRAS$^{G12V}_{166}$ are transfected with sdAb ALFA (red bars), GFP4 (purple bars) or control (grey bars)-based degraders. (B) SOCS7, (C) SPOP, (D) VHL, (E) UBOX and (F) KLHL7-based biodegraders' data. Statistical significance was determined by one-way ANOVA followed by Dunnett post-hoc tests in (B-F) (*P<0.05; **p<0.01; ***p<0.001; ****p<0.0001; ns, not significant). Error bars in (B-F) are mean ± SD of three independent biological repeats.

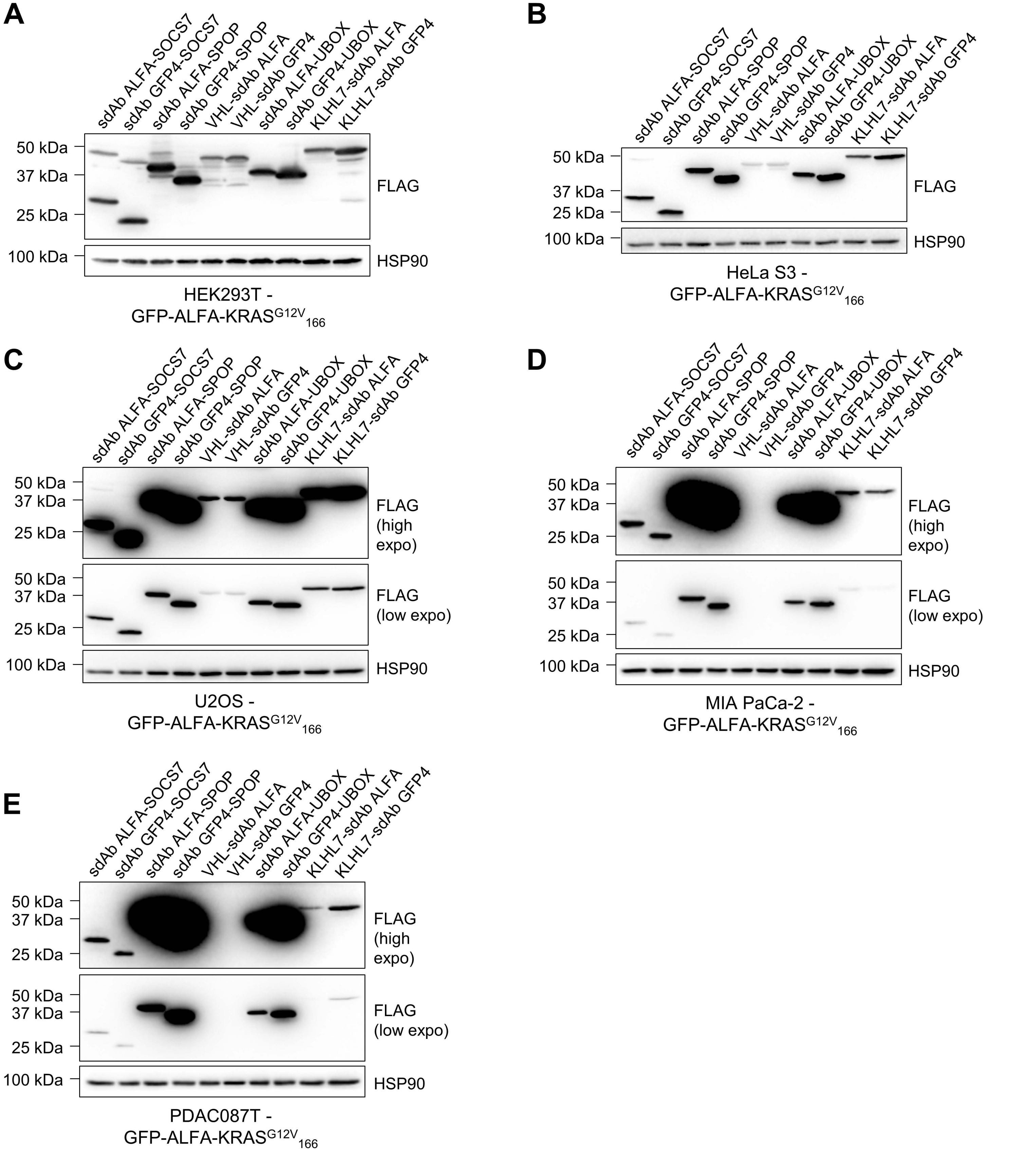

Figure S6: Control of expression of E3 ligase-based biodegraders in cell lines expressing GFP-ALFA-KRAS$^{G12V}_{166}$, related to Figure 3. Western blot analyses to control the expression level of the biodegraders (FLAG tag) from Figure 3 in HEK293T (A), HeLa S3 (B), U2OS (C), MIA PaCa-2 (D) and PDAC087T (E) cell lines stably expressing GFP-ALFA-KRAS$^{G12V}_{166}$. HSP90 is the loading control. High/low expo: high/low exposure. A representative experiment out of two independent biological repeats is shown in panel (A-E).

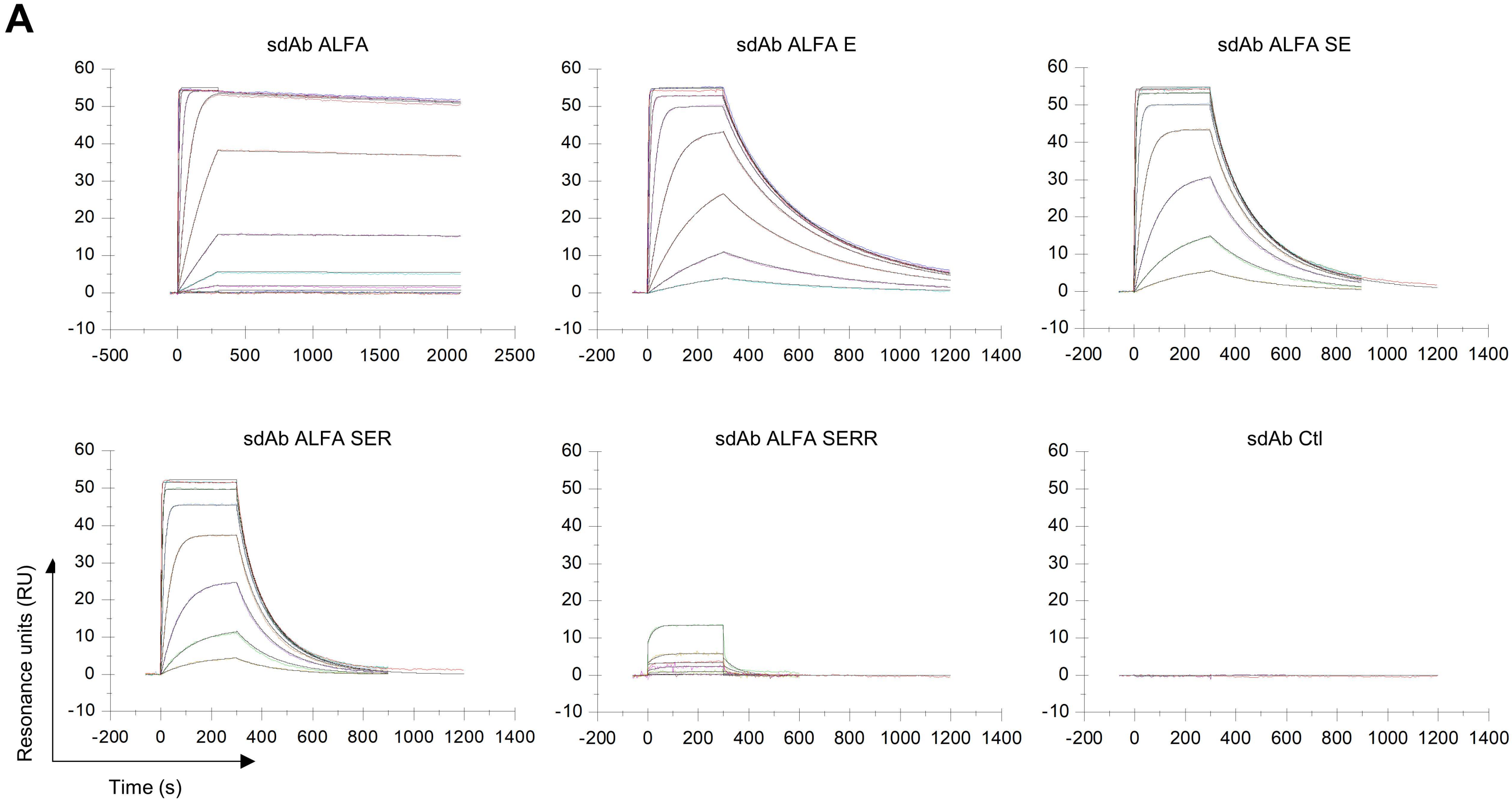

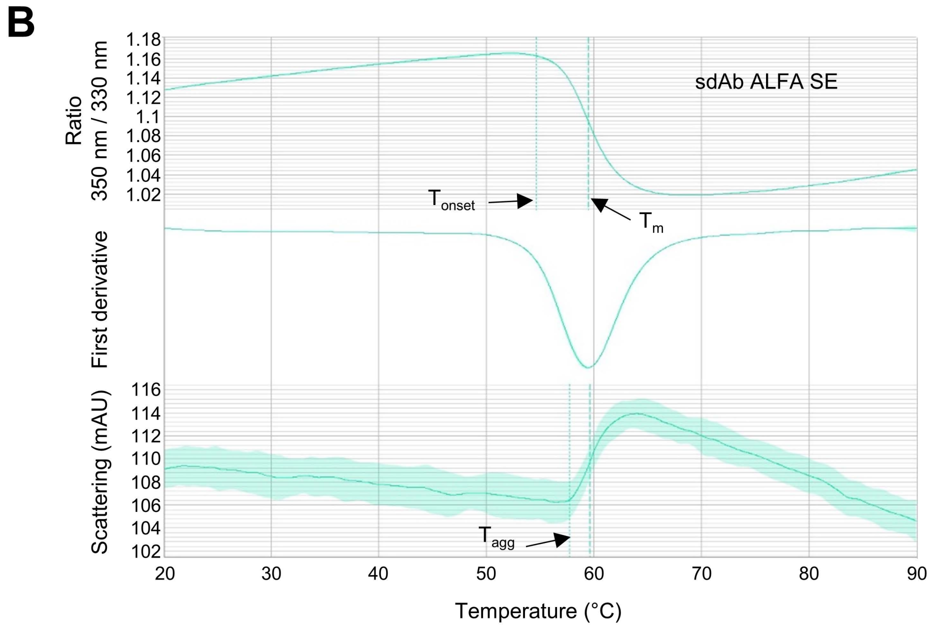

sdAb ALFA	T_{onset}	T_m	T_{agg}
WT	57°C	62°C	60°C
E	60°C	64°C	63°C
SE	55°C	59°C	58°C
SER	58°C	64°C	61°C
SERR	63°C	68°C	ND
Ctl	66°C	70°C	ND

Figure S7: *In vitro* characterisation of sdAb ALFA mutants, related to Figure 5. (A) Full kinetic analysis by SPR for each sdAb. The sensorgrams of each sdAb binding to an ALFA-tagged human Fc protein are shown. A serial dilution of sdAb ALFA (0.002 nM; 0.005 nM; 0.015 nM; 0.046 nM; 0.14 nM; 0.41 nM; 1.23 nM; 3.7 nM; 11.11 nM; 33.33 nM; 100 nM), sdAb ALFA E (0.14 nM; 0.41 nM; 1.23 nM; 3.7 nM; 11.11 nM; 33.33 nM; 100 nM), sdAb ALFA SE (0.27 nM; 0.82 nM; 2.47 nM; 2.47 nM; 7.41 nM; 22.22 nM; 66.67 nM; 200 nM), sdAb ALFA SER (0.27 nM; 0.82 nM; 2.47 nM; 2.47 nM; 7.41 nM; 22.22 nM; 66.67 nM; 200 nM), sdAb ALFA SERR (2.06 nM; 6.17 nM; 18.52 nM; 55.56 nM; 166.67 nM; 500 nM) or sdAb ALFA Ctl (500 nM) was injected over a sensorchip surface coated with ALFA-human Fc. Coloured lines: data recorded; black lines: fits used to analyse the data. (B) Thermal stability of sdAb ALFA mutants determined by nano Differential Scanning Fluorimetry (nanoDSF) method. Representative graph from nanoDSF is shown on the left panel for sdAb ALFA SE. T_{onset}: temperature at which the protein starts to unfold; T_m: temperature at which 50% of the protein is unfolded ; T_{agg}: temperature at which the protein starts to aggregate are indicated with a black arrow on the graph. The thermal stability values for each mutant are displayed on the right panel. ND: not determined because higher than 90°C.

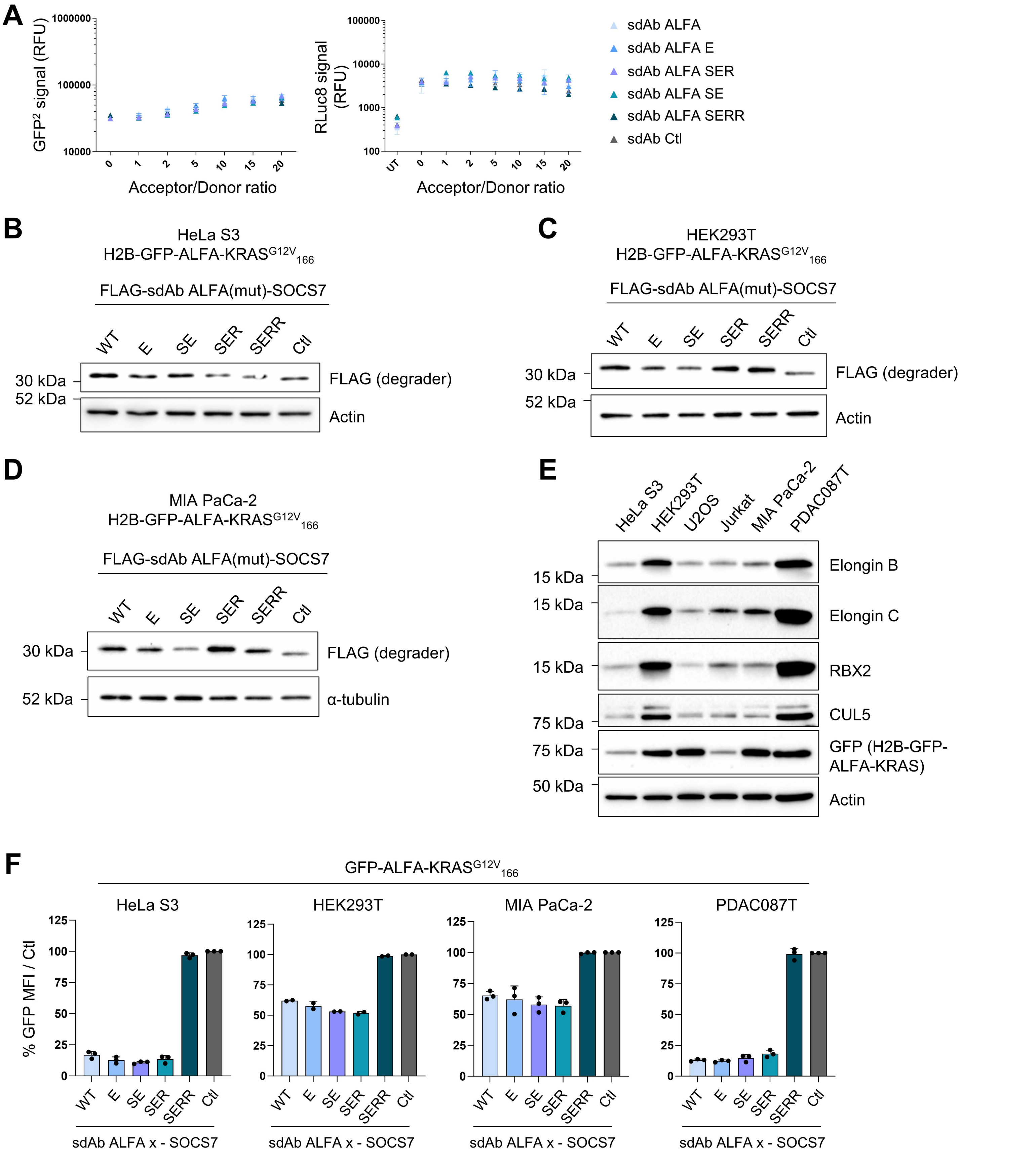

Figure S8: sdAb ALFA mutants differentially affect the degradation activity of the corresponding degrader, related to Figure 5. (A) Expression controls of the acceptors (GFP²-sdAbs, GFP² signal) and donor (RLuc8-ALFA, RLuc8 signal) from the BRET donor saturation assay shown in Figure 5C. (B-D) Representative Western blots of HeLa S3 (B), HEK293T (C) and MIA PaCa-2 (D) H2B-GFP-ALFA-KRAS$^{G12V}{}_{166}$ cells transiently transfected with sdAb ALFA mutant-based degraders. Expression of the biodegraders was detected with an anti-FLAG antibody. Actin or α-tubulin is the loading control. (E) Determination of the expression level of the different proteins forming SOCS7-based E3 ligase complex in H2B-GFP-ALFA-KRAS$^{G12V}{}_{166}$ cell lines. Actin is the loading control. (F) Normalised GFP MFI quantified by flow cytometry of HeLa S3, HEK293T, MIA PaCa-2 and PDAC087T GFP-ALFA-KRAS$^{G12V}{}_{166}$ cells transfected with sdAb Ctl and sdAb ALFA mutants fused to SOCS7. Experiments in (B-E) were performed twice. Error bars in (A, F) are mean ± SD of at least two independent biological repeats. A representative experiment out of two independent biological repeats is shown in panels B-E.

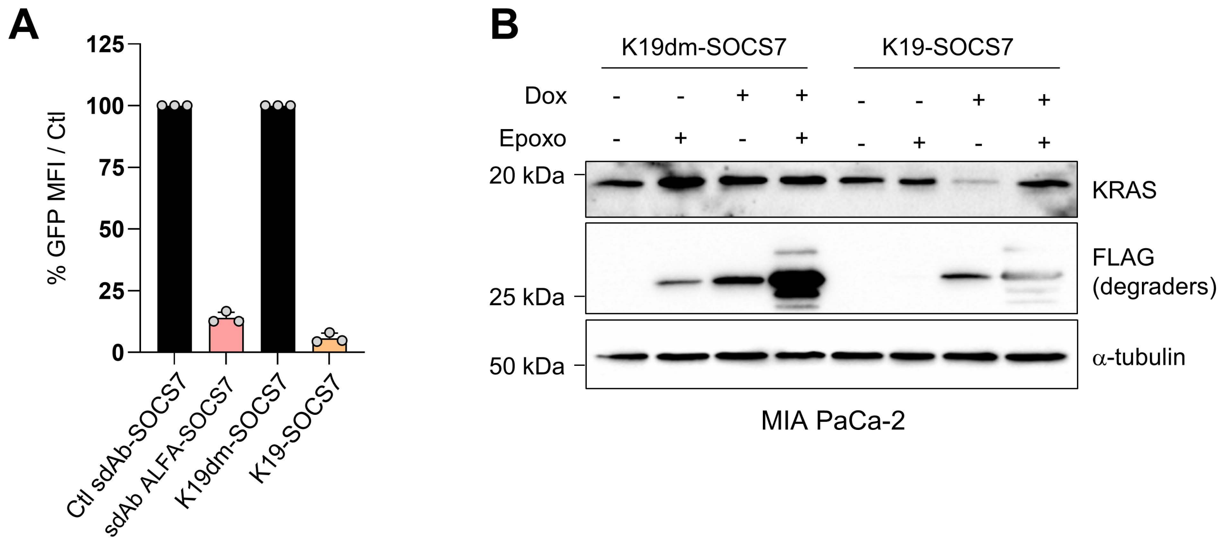

Figure S9: Characterisation of SOCS7-based KRAS degrader, related to Figure 6. (A) SOCS7 fused either to sdAb Ctl or ALFA, DARPin K19dm or K19 is transfected in HeLa S3/H2B-GFP-ALFA-$KRAS^{G12V}_{166}$ and GFP MFI in transfected cells is determined by flow cytometry and normalised to the related Ctl GFP MFI. (B) MIA PaCa-2 cells expressing K19dm-SOCS7 or K19-SOCS7 degraders were either untreated (-), treated with dox only (0.5 $\mu g.mL^{-1}$), treated with epoxomicin only (0.8 μM) or treated with dox and epoxomicin for 18 hours. The protein level of KRAS and biodegraders was determined by Western blot. α-tubulin is the loading control. Experiment in (B) was performed twice (biological repeats). Error bars in (A) are mean ± SD of three independent biological repeats.
